# Neonatal Diagnostics: Toward Dynamic Growth Charts of Neuromotor Control

**DOI:** 10.3389/fped.2016.00121

**Published:** 2016-11-23

**Authors:** Elizabeth B. Torres, Beth Smith, Sejal Mistry, Maria Brincker, Caroline Whyatt

**Affiliations:** ^1^Rutgers University, Brunswick, NJ, USA; ^2^University of Southern California, Los Angeles, CA, USA; ^3^University of Massachusetts Boston, Boston, MA, USA

**Keywords:** neural development, micro-movements, stochastic analysis, wearable sensors, inertial measurement units, accelerometers, babies, neural control of movement

## Abstract

The current rise of neurodevelopmental disorders poses a critical need to detect risk early in order to rapidly intervene. One of the tools pediatricians use to track development is the standard growth chart. The growth charts are somewhat limited in predicting possible neurodevelopmental issues. They rely on linear models and assumptions of normality for physical growth data – obscuring key statistical information about possible neurodevelopmental risk in growth data that actually has accelerated, non-linear rates-of-change and variability encompassing skewed distributions. Here, we use new analytics to profile growth data from 36 newborn babies that were tracked longitudinally for 5 months. By switching to incremental (velocity-based) growth charts and combining these dynamic changes with underlying fluctuations in motor performance – as the transition from spontaneous random noise to a systematic signal – we demonstrate a method to detect very early stunting in the development of voluntary neuromotor control and to flag risk of neurodevelopmental derail.

## Introduction

**Figure A1:**
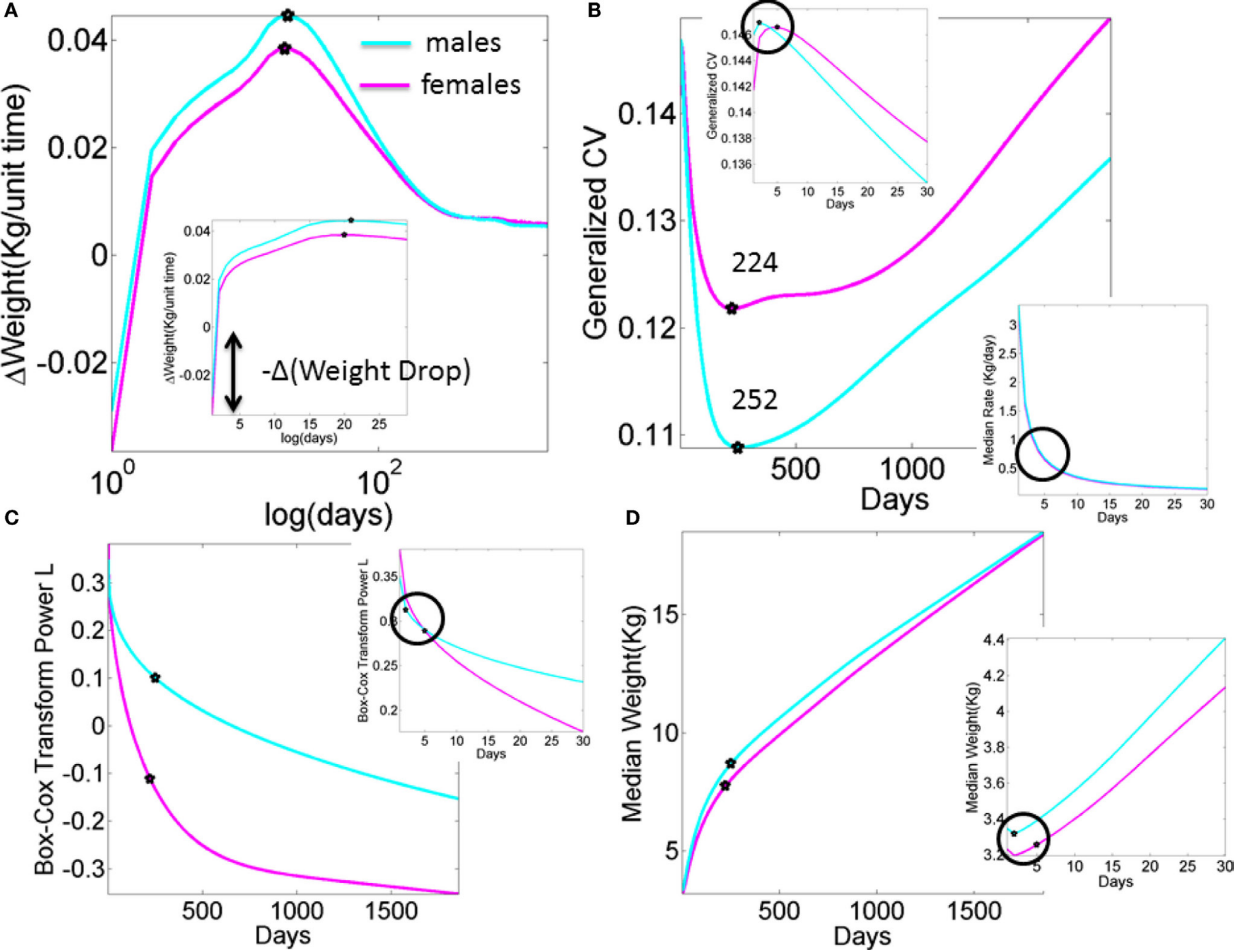
**What we are missing in our clinical assessments and basic research (data obtained from publicly available records registered to build the WHO charts)**. Stochastic, non-linear, dynamic processes clearly underlie the existing data that is at present enforced to be deterministic, linear, and static. **(A)** Progression of the change in weight day by day in male and female newborn babies according to the median weight summary drawn from 26,985 babies/summary point (13,623 girls, 13,362 boys). Babies were longitudinally tracked for 24 months upon which cross-sectional data were used to build the charts up to 5 years of age (28, 30). Inset highlights the initial drop in weight. Several inflection points in this curve have the potential to reveal additional information, particularly the first one that separates males from females in early stages of neurodevelopment. **(B)** Inflection points in the curve tracking the generalized coefficient of variation from the weight data. Female babies reach the significant minimum at 224 days, almost a month earlier than male babies at 252 days. Left-top inset zooms in the data for the first month, showing that the two groups separate in the first week after birth. Right-bottom inset shows the non-linear nature of the rate of change in median weight (zooming into the first month as well). **(C)** The skewed nature of the probability distributions underlying the physical growth parameters can be captured by tracking the *L* parameter (the Box–Cox transformation power value to enforce symmetry in skewed probability distributions (31), see also Appendix quoting the Methods paper (30) “*The assumption is that, after the appropriate power transformation, the data are closely approximated by a normal distribution”*). Notice that as in all other parameters the required transformation power *L* is different for male and female babies, denoting different families of probability distributions underlying their physical growth (in this case specifically the weight). Points mark the days when the generalized coefficient of variation reached inflection points, thus marking critical significant departures in variability in males vs. females. Inset zooms into the 1-month period to highlight the first of such inflection points as early as the first week of life. **(D)** Tracking the median weight over the first 5 years of life. Points mark the change in the underlying variability according to the inflection point in the generalized coefficient of variation. Inset zooms into the first month of life to also highlight the days when the inflection points in the underlying variability were attained in the first week of life.

**Figure 1:**
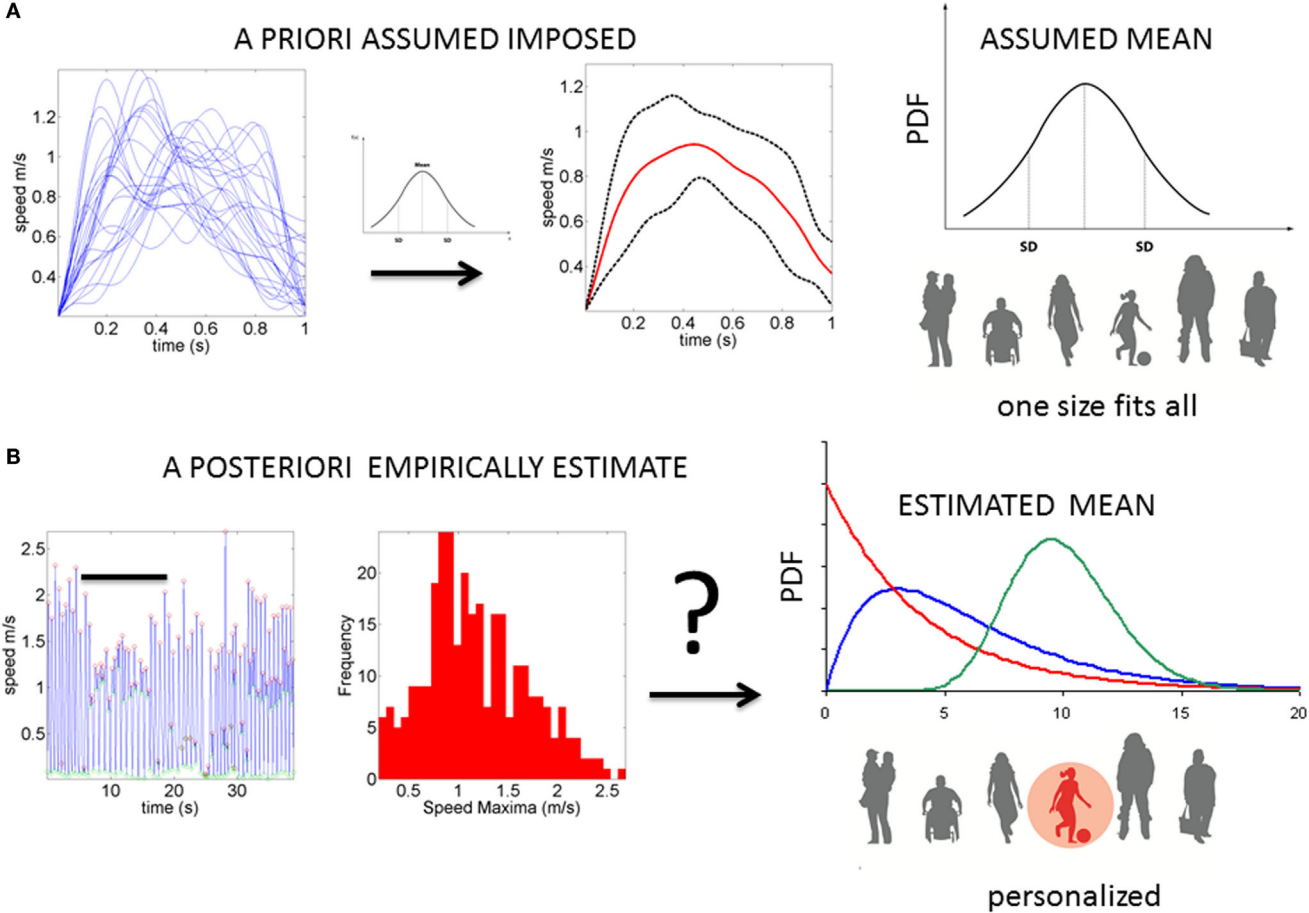
**Statistical platform for the personalized analyses of natural behaviors: (A) the prevalent “one-size-fits-all” model currently in use to analyze kinematics data**. The example shows epochs of temporal speed profiles aligned and averaged under the assumption of normality. The assumed (theoretical mean) and the assumed variance are then used to characterize the motor behavior, thus leaving out important fluctuations in motor performance (considered as noise or a nuisance). **(B)** Our proposed platform extracts waveforms of variations in motor performance and estimates the underlying family of probability distributions. This method characterizes the individual and the rate of change in PDFs as the noise (dispersion) decreases, and the signal becomes well-structured and systematic (predictable).

Neurodevelopment follows an extremely dynamic trajectory (1–4), with each infant experiencing a range of unique changes, driven by both the infant and their own environment. During the early stages of neurodevelopment, the infant’s body and head grow at an accelerated rate (e.g., see Figure A1), and the nervous systems of the infant must rapidly develop in tandem to adapt to, and to compensate for, these changes. Due to the variable nature of biological systems, these day-to-day fluctuations in physical growth follow a non-uniform, non-linear process, with some babies changing at slower rate than others at certain times. Likewise, the fast-changing nervous systems underlying the fast-growing physical body must develop rapidly to create the foundation for purposeful controlled actions. In the face of such highly variable neurodevelopmental processes, it may be important to switch from the “one-size-fits-all” model currently in use (Figure 1A) to a personalized statistical approach (Figure 1B). In particular, the use of a personalized approach is more adequate to individually fit, and thus “capture,” the true nature of the adaptive processes of the early stages of a newborn’s life.

The physical growth of the baby’s body can be easily monitored; indeed, this is often tracked with regular visits to a pediatrician. The pediatrician will utilize a range of standardized tools, such as standardized population growth charts, that have been created and produced by both the Center for Disease Control (5) and the World Health Organization (6) to track progress. Although not intended as diagnostic tools, pediatricians often use such charts to infer general aspects related to neurodevelopmental progress. Yet, such methods are imprecise (7, 8) (see Appendix). This imprecision may mask early signs of neurodevelopmental delay or difficulty. Besides physical growth, proper neurodevelopment includes the emergence of voluntary control of the developing brain over the changing body. One way to track the maturation of this form of neuromotor control more precisely is by longitudinally registering physical motions and examining the emerging trends of the individual’s physiological signatures. More specifically, one can track the baby’s development of neuromotor control by statistically characterizing the stochastic fluctuations in motor performance as the nervous system adapts to the accelerated physical growth. Indeed, as the bodily rhythms evolve and transition from spontaneously random to well-coordinated movements, the corresponding changes in their statistical patterns may reveal the signatures of voluntary control.

The relevance of bodily rhythms and the infant’s ability to self-organize and synchronize them with external environmental rhythms should perhaps be more seriously considered in contemporary pediatrics. Indeed, it has been reported that newborn infants naturally entrain their bodily rhythms with those of the adult’s speech (9); an ability that is compromised in individuals that go on to receive a diagnosis of a neurodevelopmental disorder (10). Related perinatal research points at the intertwined relations between respiration, sucking patterns, and speech (11, 12). Such work highlights the importance of well-functioning orofacial sensory–motor structures to scaffold the production of motoric rhythms (11, 13), the later emergence of spoken language abilities, and their potential role as precursors of other cognitive and social capacities.

**Figure 2:**
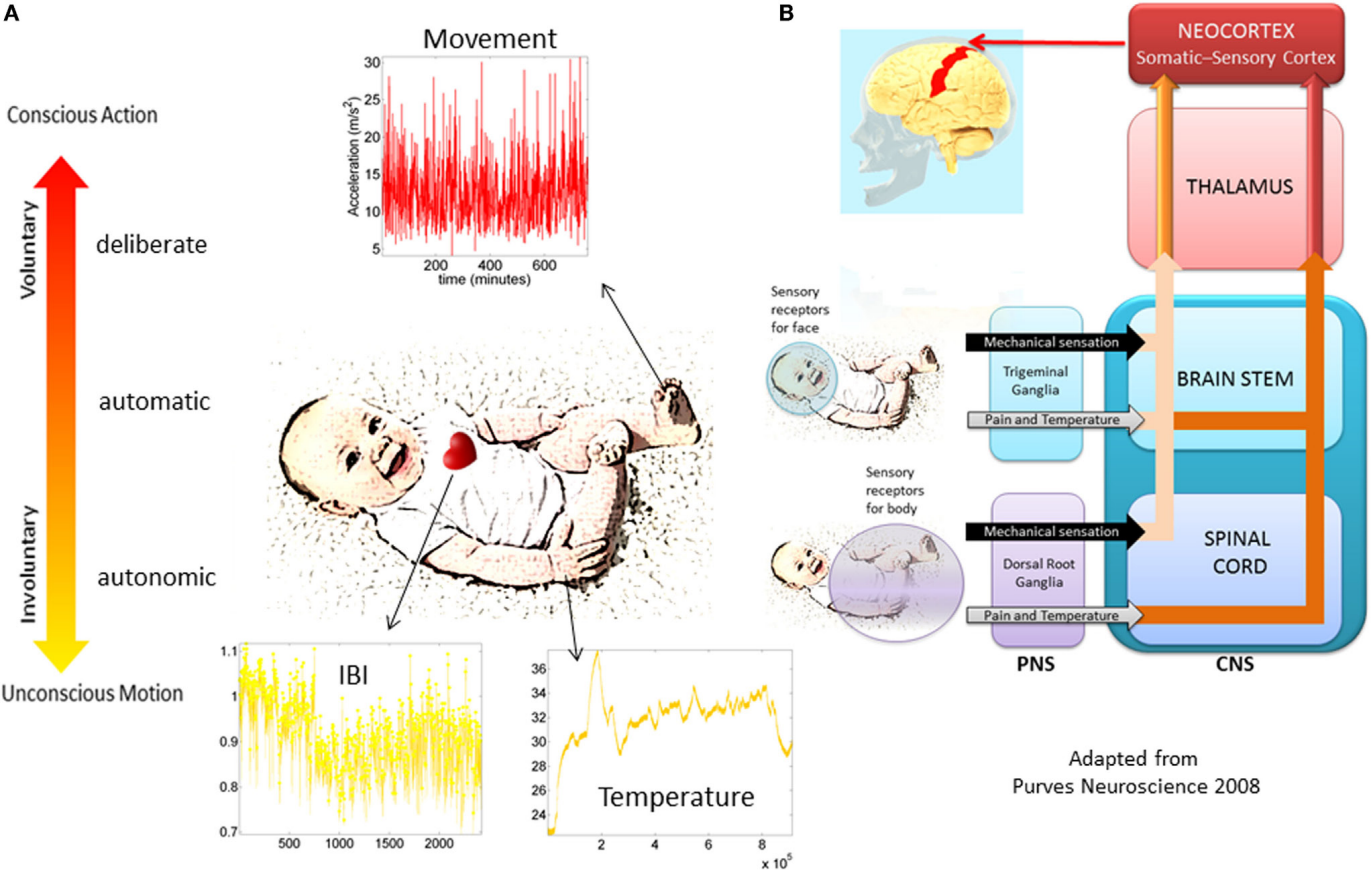
**Proposed taxonomy of neuromotor control**. **(A)** Different layers of control across the nervous systems appear in phylogenetic order from the bottom up. Information in each level can be registered non-invasively with modern instrumentation. The maturation of each layer of control transfers to the next layer as the baby develops. Their intertwined balance scaffolds the development of voluntary control of the bodily rhythms at will (volition). This (we posit) is a necessary ingredient to develop cognitive abilities. **(B)** Schema adapted from (16) showing the autonomic and sensory–motor systems and the necessary interactions for further neurodevelopment and the well-functioning of all aspects of social interactions.

The bodily rhythms are under different levels of control and involve different structures throughout the nervous systems with different phylogenetic order of maturation Figures 2A,B. Such orderly anatomically structured and layered systems begin with autonomic functions and reflexes that soon evolve in the neonate; or not as in the cases where neurodevelopmental disorders were later found (14, 15). Indeed, the proper functioning of autonomic systems and reflexes from an early stage of life seem to be critical to survive, while also providing the foundations for the development of autonomy and self-control of the peripheral nerves by the central cerebrocortical structures. We believe that at these early stages important organizational maps involving sensory inputs and nervous systems’ reactions to external and internally generated stimuli begin to form. Gradual adaptation of these maps may eventually lead to the formation of new modifiable-on-demand maps of sensory consequences of impending actions. As such, monitoring the early evolution of bodily rhythms in the neonate, as they transition from spontaneous random noise to detectable signals may be relevant. In this sense, describing the ranges of variability of some of those rhythms may help characterize normative vs. atypical trends of neonatal adaptation of nervous systems performance in response to physical bodily growth (Figure 2B).

The various degrees of variability associated with the various layers of neuromotor control in Figure 2A have different noise-to-signal ratios (17–19). This type of information, echoed back to the brain through reafferent loops (20), may play a critical role in early stages of neurodevelopment. More specifically, we posit that the rates of change in the stochastic signatures of bodily rhythms, particularly in the neonatal stages of development, may help forecast the adaptive capacity of the developing nervous systems. As such, it is our hypothesis that the degree of congruence between the rates of adaptation of noise-to-signal transitions in kinematics variables reflecting higher levels of control and those of physical growth must be a good indicator of typical neurodevelopment in the newborn. The failure of this congruence to appear may flag risk of neurodevelopmental stunting. Thus, this work offers an operational account providing empirical evidence for this hypothesis.

## Materials and Methods

### Participants

Twelve infants with typical development (8 females, 4 males) forming a control group (CT) and 24 infants (9 females, 15 males) pre-labeled clinically at risk (CAR) for developmental delay participated. Infants with typical development were from the Portland, OR, USA, metropolitan area. Infants at risk for developmental delay were from the Los Angeles, CA, USA, metropolitan area and were identified as at risk according to the clinical definition of the state of California (21). This group includes, for example, infants born preterm, with traumatic birth experiences, or with congenital heart defects. From this CAR group, it is anticipated that approximately half will have poor neuromotor outcomes (a diagnosis of developmental delay at 24 months), while half will have good neuromotor outcomes (no diagnosis of developmental delay at 24 months). Our target developmental period was birth to walking onset (defined as onset of three independent steps). Infants with typical development started the study between 1 and 8 months of chronological age while infants at risk started between 1 and 15 months chronological age. All infants except for two were measured three times with ~2 months between visits, spanning 5 months of their early development. These two infants were only measured twice as they started walking before the third scheduled measurement. The study was approved by the Institutional Review Boards (IRB) of Oregon Health & Science University and the University of Southern California. Parents signed an informed consent form for their infants’ participation. Rutgers University IRB approved data sharing agreements to properly examine the de-identified data.

### Data Collection

At each visit, the Alberta Infant Motor Scale (AIMS) (22) was administered to the babies in order to quantify motor development status. Additionally, physical growth parameters were registered, including measurements of body length, body weight, and head circumference. Inertial measurements units in wearable sensors (Opals, APDM, Inc., Portland, OR, USA) were placed in each leg using knee socks or custom leg warmers with pockets (Figure S1A in Supplementary Material). If knee socks were used, sensors were firmly attached with Velcro^®^ to the bottom layer knee sock, proximal to each ankle joint and secured with a second knee sock. They synchronously collected triaxial acceleration, triaxial gyroscopic motions, and temperature at 20 Hz continuously for 8–13 h. Visits took place in the morning, and the sensors remained in place during all typical activities of the day until bedtime; this was 8–13 h after placement, when parents were instructed to remove them. Data were stored in the sensor’s internal memory and downloaded later for analyses. The data analyzed here include all hours of continuous motions.

### Statistical Analyses

The time-series data from the inertial measurement units (IMUs) attached to the legs of the infants were analyzed using new techniques that estimate the family of probability distributions best characterizing the continuous random process underlying the spontaneous movements of the babies. These time series from the IMU-wearables are transformed into time series of the acceleration peaks. A waveform derived from these time series is then used to represent a continuous random process under the general rubric of Poisson random process (PRP). To be more precise, we treat the spikes in acceleration as spike trains of random amplitudes and random times. To model them, we build on previous research whereby the amplitudes and inter-spike interval times are modeled as independent and identically distributed (iid) random variables following a Gamma distribution (18, 19, 23, 24). As such, the spike trains are the input to a Gamma process and the empirically estimated Gamma parameters are dynamically tracked on various parameter spaces to uncover self-emerging patterns. In particular, on the Gamma parameter plane, we use the median values of the two parameters of interest (the shape and the dispersion of the distributions) to define quadrants. We track the jumps of the parameters from the quadrant of low dispersion (low noise-to-signal ratio, NSR) and symmetric shapes [denoted right lower quadrant (RLQ)] into the quadrant of high NSR and skewed shapes with limiting case of the most random (memoryless) exponential distribution [denoted left upper quadrant (LUQ)].

By tracking the frequency and amplitude of the shifts in probability distribution functions between the LUQ and the RLQ, these data-driven methods automatically determine the individualized transitions from spontaneous random noise to well-structured, systematic signals in relation to the rate of physical growth of each baby. The detailed explanation of the methods and figures illustrating them can be found in Supplementary Material. Their use has been amenable to computational tractability of Big Data collected over the course of several hours (17). Moreover, they facilitate statistical inference and further interpretation of the results in light of clinical scores.

### Summary or Roadmap of the Analyses

There are three types of analyses presented in the paper. First, we compute the ensemble behavior of each clinical group of the typical babies and the group at risk according to their *a priori* given clinical label (CAR). We report outcomes from non-parametric statistical tests. Since the two groups had disparate sizes (CT 12) and (CAR 24), we randomly chose 12/24 with no replacement until we exhausted the CAR group and report the average *p-value* obtained from the non-parametric rank-sum Wilcoxon test or the Kruskal–Wallis test comparing equal-size CT and CAR groups. Using these clinically pre-labeled groups, we examined the temperature fluctuations in each group and the physical growth parameters reported for each baby in each group. We also examined the stochastic signatures and their shift or lack thereof. The results from these analyses are presented in the Figures S6 and S7 in Supplementary Material.

The second set of analyses was performed on the whole ensemble in *data-driven* mode, i.e., without considering the clinical labels (CT and CAR). The motivation here was to unveil automatically self-emerging clusters based on the objective physical growth data in order to guide the classification of the underlying noise-to-signal data extracted from the motion sensors registering leg movement rhythms. The resulting data-driven groups were then examined to see which babies came from the clinically pre-labeled groups born with or without complications.

This second layer of analysis was completed through a number of steps. First, we obtained the physical parameters for each individual baby (i.e., body length, body weight, head circumference) along with the parameters rate of change for each individual. This rate of change was computed by dividing the value of the parameter on each visit by the number of days since birth until the day of the visit. Examining the rate of change of the parameters rather than the absolute number of days since birth was important because the babies in the CAR group did not start the study at the same time as the babies in the CT group. Similarly, we examined the rate of change of the AIMS total scores. The median for each of the three dimensions of the rate of change in physical parameters was calculated and used to rank the data. Using the median criterion is a standard procedure and is most appropriate for skewed data, such as that generated by the incremental data used here. The three-dimensional median vector determined the cutoff of the first group of babies, those babies with the highest rate of change in each of the parameters of physical growth. This group had the highest median value for body length, weight, and head circumference. The same operation was performed on the remaining group and a second subgroup obtained, again ranked according to the median cutoff of that remaining group. This median-cutoff selection was completed once more, resulting in a total of four ranked groups. Second, a Delaunay triangulation (25) was performed on the scatter of each group to draw the corresponding surfaces and examine the underlying changes in the amplitude and the frequency of the noise-to-signal transitions. This individualized analysis also entailed examination of the rates of change in total AIMS scores so as to gain insight into the motor readiness of each ranked group and to help visualize the physical growth rate of change data.

The third set of analyses comprised the use of the stochastic signatures of acceleration obtained within each temperature range. The averaged Δ*N* in a visit was expressed as a rate of change (by dividing the Δ*N* by the number of days since birth until the day of the visit of each baby). This quantity was expressed as a function of the averaged Δphysical growth parameter, also taken as a rate of change across visits. The latter included averaged Δbody-length across visits (centimeters per day); Δbody weight across visits (kilograms per day); averaged Δhead circumference across visits (centimeters per day). This analysis automatically yielded a group of babies statistically at high risk (denoted HR). The median statistic to rank that data was the only heuristic applied. This method was appropriate given the skewed nature of the families of probability distributions underlying both the rates of change in physical growth and the rates of change in the noise-to-signal transitions of the motor fluctuations data. We denoted babies in the first ranked group typically developing data-driven TD, based on the statistical ranking (rather than on the clinical criteria). Those in the second and third groups merged into the partially at risk group (PAR) and the remaining last ranked group HR. The data-driven PAR group was comprised by babies ranked 2 and 3 according to the median-ranking criterion for the rate of change in physical growth.

## Results

### Babies Automatically Group According to Their Rates of Change in Physical Growth

**Figure 3:**
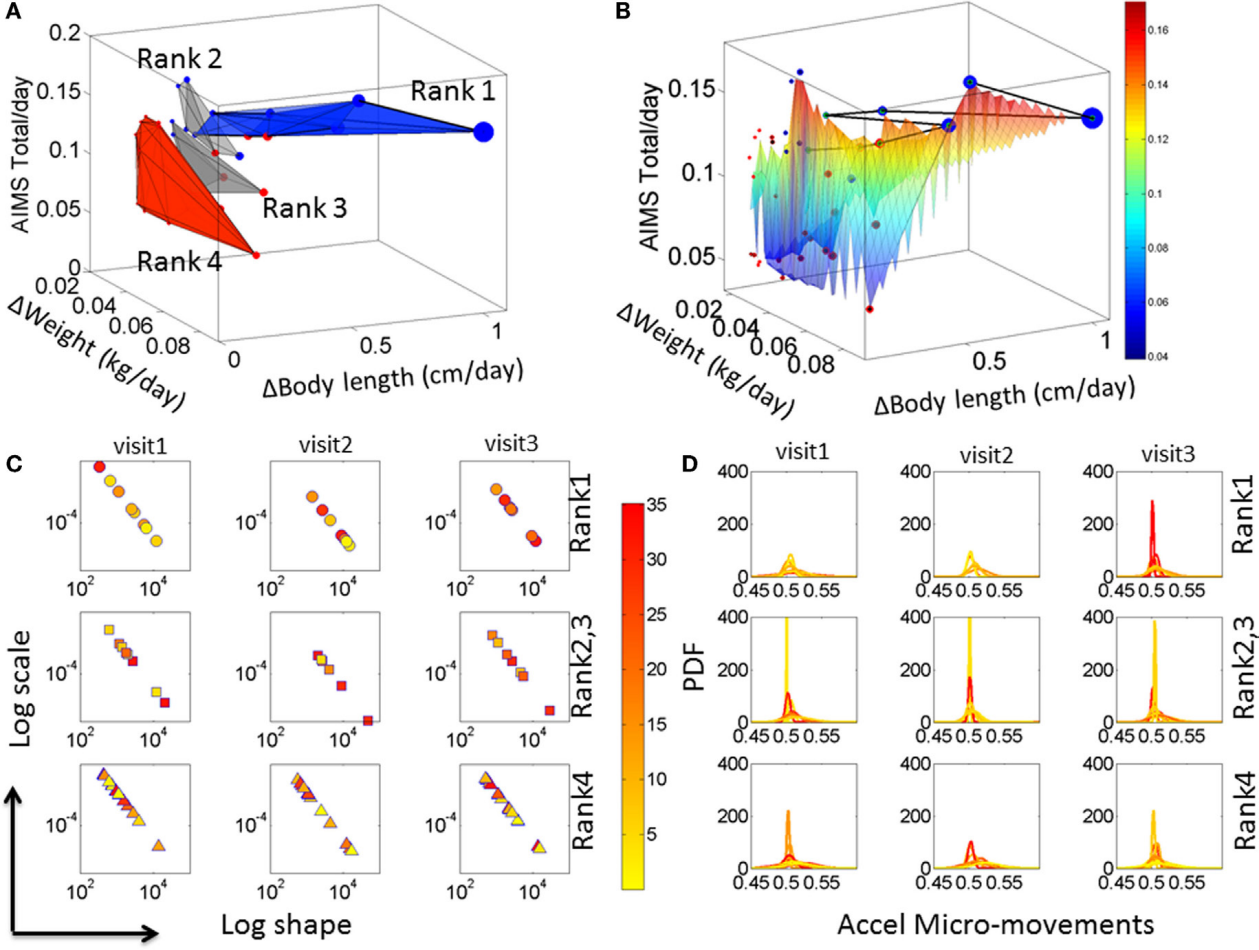
**Automatic clustering of all babies obtained from the median-ranking of the rate of change data drawn from physical growth and fluctuations in motor performance**. **(A)** The mean value of the rate of change in physical growth across visits was obtained for each one of the 36 babies. Then the median of each physical growth parameter was obtained for the entire group (see axes labels). Babies were median-ranked according to the rates of physical growth Rank 1–4 (see Materials and Methods). **(B)** A Delaunay triangulation surface was used to fit each of the four ranked scatters, whereby the size of each circle in the scatter denotes the rate of change in head circumference. The surface is further colored by the rate of change in AIMS score per days since birth to visit. The black line indicates the Group 1 babies sorted by the AIMS score from lowest to highest. The color bar denotes the average ΔAIMS score across visits. **(C)** Median values of the estimated shape and scale parameters (as in Figures S3B,C in Supplementary Material) colored according to the temperature regime of the micro-motions in the acceleration and **(D)** the corresponding PDFs for each child. Color bar shows the temperature values.

Iterative ranking of the median values attained data-driven clustering across all rates of change in physical growth parameters (see Materials and Methods). This yielded four groups sorted from highest to lowest rate of change in all three physical growth parameters. Babies in the first group were the most advanced in their rate of growth. Babies in the last group were those with the slowest rate of change, i.e., growth. Delaunay triangulation on the scatter of each median ranked group was used to determine the surface best fitting each cluster. Figure 3A illustrates the four clusters (referred to as Rank 1–4) arranged using the Delaunay surfaces with the *x*-axis representing the rate of change in weight (kilograms per day), the *y*-axis representing the rate of change in body length (centimeters per day), and the *z*-axis representing the rate of change of the total AIMS scores per day. The figure also contains the information on the rate of change in the head circumference (centimeters per day) plotted as the size of the marker. Markers colored in blue represent CT babies, while markers colored in red are from CAR babies.

Figure 3A indicates that the surface representing the babies with the fastest rate of physical growth is oriented differently from the other surfaces representing the other clusters of babies. Indeed, when examined with the clinical labels, the last cluster representing the lowest rate of change of physical growth across all parameters was composed primarily of CAR babies and one CT baby. This data-driven cluster is oriented nearly orthogonal to the Rank 1 surface (ranked according to the median ranking methodology) and is comprised of statistically at HR babies. The data-driven PAR babies are those in Rank 2 and Rank 3, whereas Rank 1 are denoted the data-driven TD group. The compositions of each group along with the ranges of the rates of change of physical growth for each group and the temperature ranges are reported in Table S3 in Supplementary Material.

To help visualize the scatter data, a surface was fit across all points in the scatter. The surface was colored according to the rate of change of the AIMS total score. This is shown in Figure 3B with the line connecting the babies in Group 1 (Ranked 1) traced from left to right in the order of the rate of change of the AIMS total score. In this panel, babies with the higher rate of growth in head circumference (represented by the size of the marker) lead the group. The two babies at the tail of this path are two CAR babies that made the ranked median cutoff above the rest and into the Rank 1-TD group. These two babies (twins) began this process with delayed development following premature birth. However, they seem to have “caught up,” which is appreciated at the rate of change level. In contrast, when examining the absolute value of the parameters only, these two babies seemed similar to the other CAR babies.

These results prompted us to assess the fluctuations in motor performance and the noise-to-signal transitions underlying these rates of physical growth. Figure 3C shows the results from these analyses as we estimated the signatures of neuromotor control by integrating output in motor and temperature fluctuations. The panels of Figure 3D show the estimated probability distribution function corresponding to the Gamma parameters on the log–log Gamma plane in Figure 3C. Each dot represents the median values of each baby across a large number of estimated parameters from 8 h of continuous recordings (see Supplementary Material). The color (as in Figures S3 and S4 in Supplementary Material) represents the median temperature associated with each set of motor fluctuations. Babies in the Rank 1 group are found to have a trend toward higher temperature values as time progresses, which due to the design of this sensor technology is indicative of higher levels of actively self-generated motions (draining more battery energy than passive motions and therefore heating the sensor more). These active motions are also mirrored in shifted physiological signatures that gradually move down and to the right on the Gamma parameter plane. From visit to visit, these shifts in temperature values and Gamma parameter values are more pronounced in the Rank 1 than in the Rank 4 groups. Indeed, in the Rank 1 group, from visit 1 to visit 3, the drop in noise from higher to lower and the change in shape from skewed to symmetric are significant (rank-sum Wilcoxon test *p* < 0.01) but not so in Rank 4 babies (*p* < 0.9). Note: the right panel of the figure shows the estimated PDFs corresponding to the Gamma parameters in the right panel.

We next assessed these signatures as rates of change (dividing them by the number of days since birth) and to quantify the frequency of noise-to-signal transitions that each baby underwent each visit, and the average transitions across visits for each statistically ranked Group.

### High Inner-Quadrant and Inter-Quadrant Frequency of Noise-to-Signal Transitions Mark Typical Neurodevelopment

For each of the individual members of each ranked group, we examined the minute fluctuations in motor performance. To this end, we used the methods described in Figure S5 in Supplementary Material and characterized the stochastic signatures of these fluctuations as they transitioned from the LUQ to the RLQ of the Gamma parameter plane, i.e., the probability distribution functions transitioning to low noise-to-signal ratio and distribution shapes shifting toward symmetric distributions.

**Figure 4:**
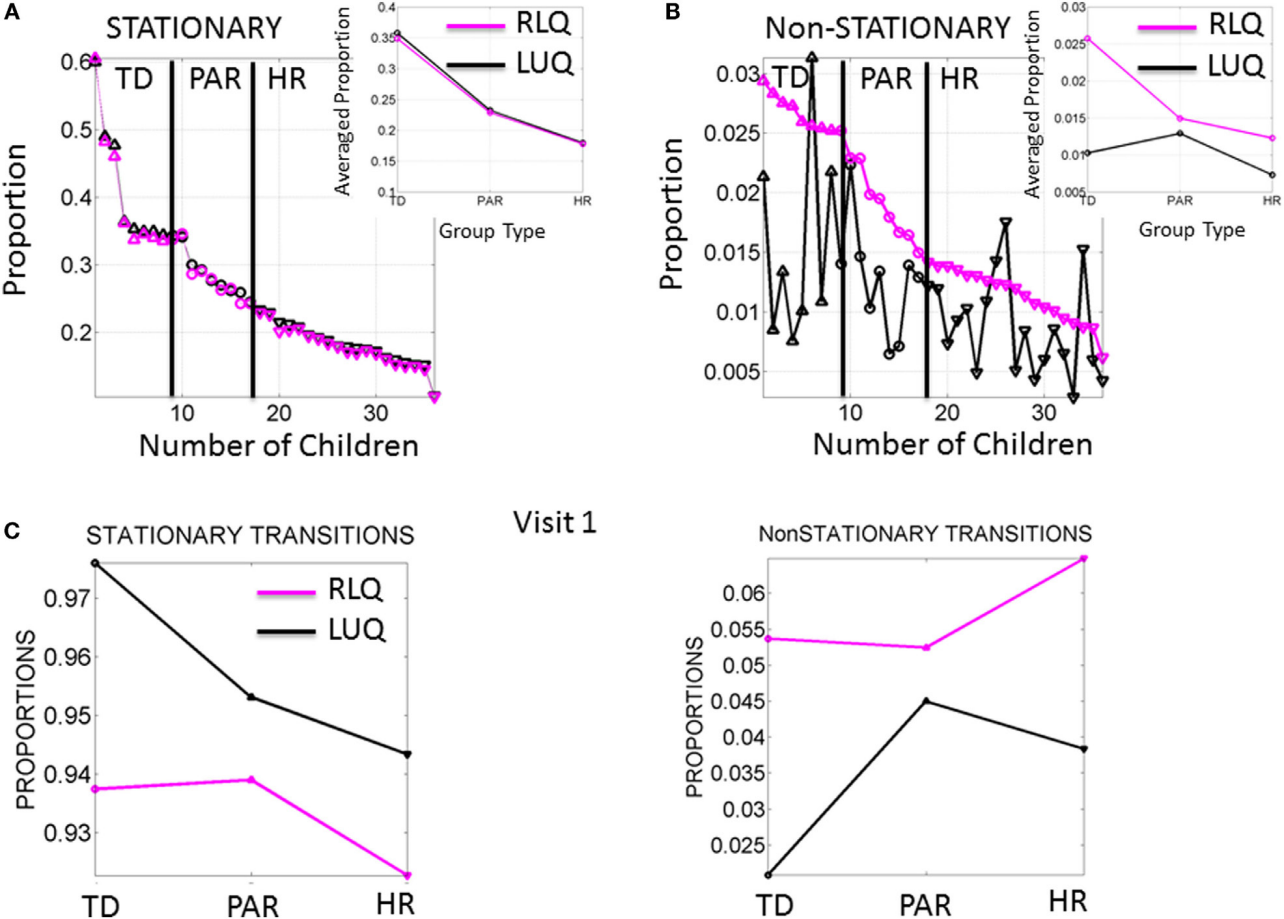
**Frequency of noise-to-signal transitions distinguishes babies at high risk from typically developing babies**. **(A)** Stationary (inner-quadrant) transitions sorted according to the proportion of times fluctuating within each quadrant before crossing to the other quadrant. Each dot represents a baby (up triangles are TD, circles are PAR and down triangles are HR). Inset is the median across each group. **(B)** Non-stationary (inter-quadrants) transitions sorted according to the proportion of times crossing across quadrants from the RLQ to the LUQ. The same index used to plot the opposite direction of transitions shows higher variability when crossing from the LUQ the RLQ. Inset shows the median values/group. **(C)** Median values of noise-to-signal transitions/group during the first visit already distinguish the groups in both the stationary and the non-stationary cases.

The signatures of fluctuations in motor performance of the babies in Rank 1 group transitioned far more frequently from higher to lower noise and from highly skewed to more symmetrically shaped PDFs than those of babies in the other groups. This is depicted in Figure 4A for the stationary case where the transitions remain within the LUQ or within the RLQ. In this case, the babies are ranked according to the proportion of times that their signatures remained in a “steady-state” within one quadrant or the other. The inset of the Figure 4A depicts the three data-driven groups from the median ranked parameters of physical growth in Figure 3A. Specifically, babies in the first group (data-driven TD) have the highest proportion of remaining steady in the LUQ or the RLQ on average. The data-driven PAR group falls intermediate to data-driven TD and HR groups. The HR group has the lowest proportion of “steady-state” in LUQ or RLQ.

The non-stationary noise-to-signal inter-quadrant transitions shown in Figure 4B right panel were characterized by multiple shifts between the LUQ and RLQ. Their proportions also showed – for each individual baby – patterns that distinguish the TD from both the PAR and HR groups. Here, the transitions to the RLQ were ranked by proportion (individualized rates for each baby obtained relative to the overall total frequency of transitions of each baby) and systematically decreased according to the group type. In contrast, the transitions of the LUQ were highly variable across the three groups. The inset shows the differences in average proportion taken across all members of each group. Here, the frequency in dynamically transitioning across the quadrants clearly separates those TD babies from the PAR and the HR babies.

Figure 4C shows the median values of the stationary and non-stationary transitions for the first visit. This result shows that differences in neuromotor developmental trajectories associated with the rates of change of physical growth can be detected within the first months of infancy.

### Steady Rate of Growth in Physical Changes Corresponding to Steady Rate of Change in Transitions from Noise-to-Signal Mark Typical Neurodevelopment

In addition to the frequency in noise-to-signal transitions, the magnitude of the maximal shift between the LUQ and the RLQ was significantly higher in the data-driven TD group of babies (*p* < 0.01 rank-sum Wilcoxon test). To further quantify this, we aimed at finding a relationship between the maximal noise-to-signal transitions per day and the change in physical growth parameter per day across the abovementioned three groups. The scalar denoting the average across the visits for each of the neuromotor control and physical growth domains was plotted for each baby on a parameter plane and a line through the scatter corresponding to each group was fit.

**Figure 5:**
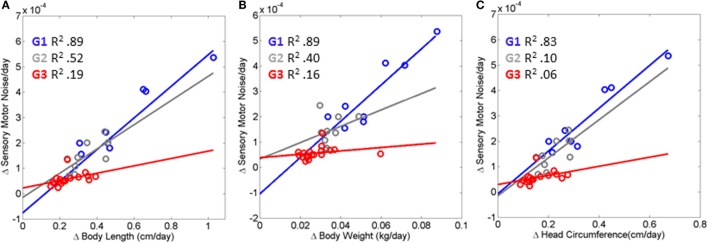
**Index of risk uncovered to automatically flag neurodevelopmental derail or stagnation very early on**. **(A–C)** Linear relation is found between the rates of change in physical growth and the rates of change in the neural control of movements measuring the maximal amplitude of the transition from the LUQ to the RLQ (see main text reporting the goodness of fit parameters for each scatter). The relation degrades and the slope flattens as the baby’s body grows slower and does not undergo changes in the signatures of sensory–motor noise derived from fluctuations in motor performance. HR, red scatter (Rank 4 Group G3); PAR, gray scatter (Rank 2, Rank 3 G2); and TD, blue scatter (Rank1 G1).

Figure 5 shows the results of this fitting for each of three groups (TD, PAR, and HR). The TD (Group 1 comprised of Rank 1 babies) displayed linear relation with positive slope (*R*^2^ 0.89, 0.89, 0.83) for body length, weight, and head circumference, respectively, and change in sensory–motor noise – i.e., transitions. This trend was followed by a weaker relation (*R*^2^ 0.52, 0.40, 0.10) between parameters for the PAR group – Group 2, formed by Rank 2 and Rank 3 babies. The babies in the HR group – Group 3 formed by Rank 4 babies, characterized with the slowest rates of change in growth – had a flat slope (*R*^2^ 0.19, 0.16, 0.06) indicating stagnation in the rate of change of noise-to-signal transitions toward motions under volitional control. These HR babies, classified as such with respect to the rate of change of physical growth, are also at risk of neurodevelopmental derail because noise-to-signal transitions were absent or did not evolve within each of the three visits. When examined longitudinally, their rates of change were stagnated in both frequency and amplitude. They did not evolve these metrics of neuromotor control from visit to visit.

## Discussion

This work set to uncover an index of risk for the early detection of neurodevelopmental stunting in newborns. To this end, we examined 36 newborn babies longitudinally, over the span of 6 months and 3 visits. We performed statistical analyses of two groups with an *a priori*-given clinical label of control (CT) vs. CAR groups. The CT group was composed of babies typically born full-term without complications and the CAR group was composed of babies with complications at birth. These analyses confirmed marked statistical differences on the classical growth parameters and other parameters from wearable sensors hinting at less overall motion in the babies at risk. In addition, *data-driven* analyses were completed, without the *a priori* given clinical labels to identify any babies born without complications that do undergo subsequent stunting in neurodevelopment.

For each individual baby, we examined the rates of change in physical growth in tandem with the rates of change in the empirically derived statistical signatures of neuromotor control. A gradient of values emerged across the cohort characterizing the relationship between physical growth and neuromotor control development. At one end of this gradient were babies that grouped according to congruent rates along these two dimensions. This group had a higher regression coefficient (adjusted *R*^2^ closer to 1) between the rate of change of physical growth parameters and those characterizing sensory–motor control than babies with stunted neurodevelopment – characterized by the group that emerged at the opposite extreme of this quantification process. Indeed, babies in the group that demonstrated neurodevelopmental stunting did not significantly change from visit to visit. Unlike the cluster of TD babies with congruent rates of change in both of these parameters, the group at HR did not show correspondence between the evolution in the stochastic signatures of acceleration and those of physical growth. Specifically, the transitions from high noise-to-signal ratios into well-structured signals showed very little change from visit to visit for the group at HR. Likewise, the rate of physical growth in this group of babies was significantly slower than that in the developing group (as shown in Figures 3A,B).

**Figure A2:**
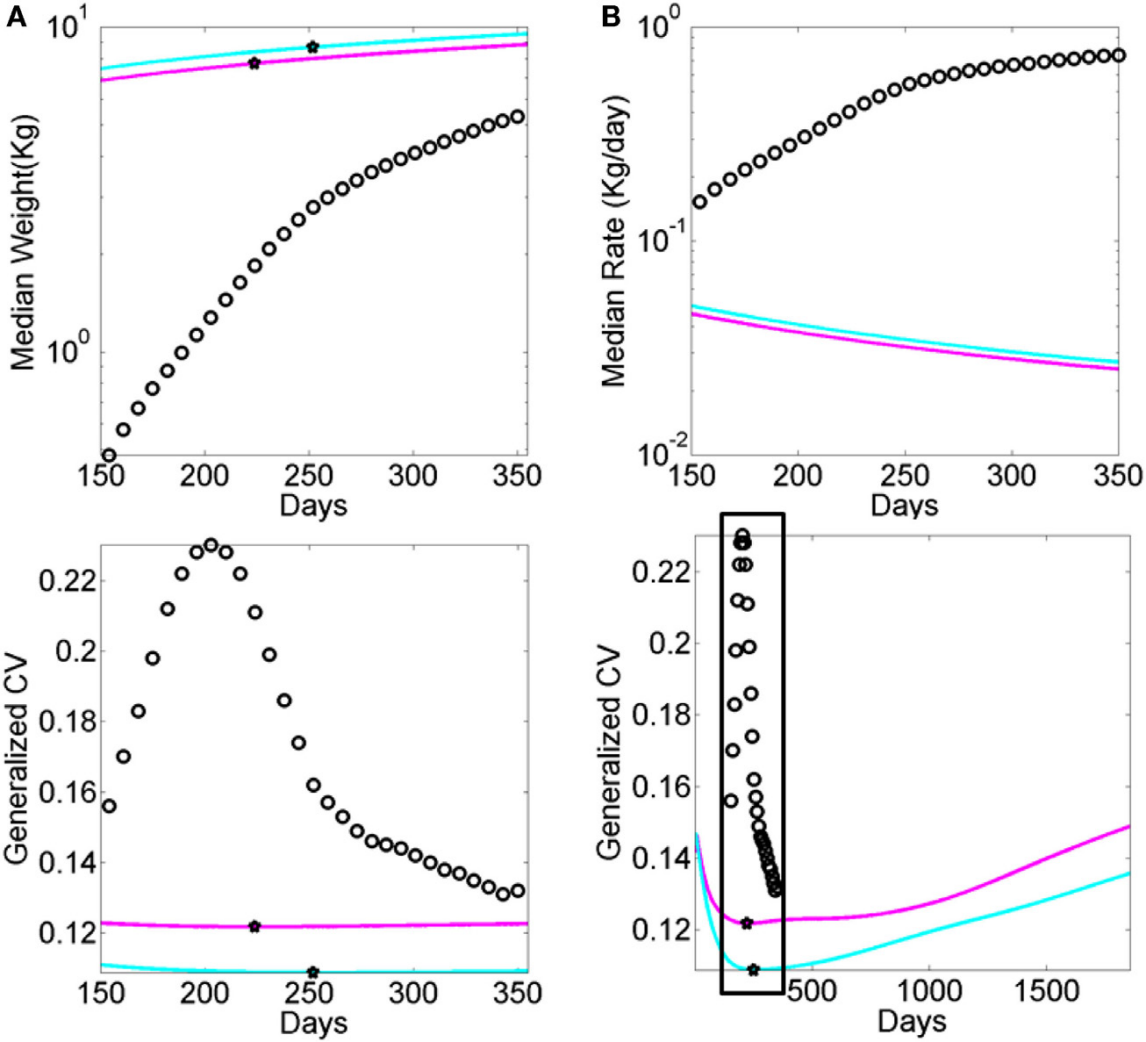
**Data from premature babies superimposed on the data from typical babies obtained from the WHO growth charts (weight parameter in Figure A1)**. Each circle represents the summary median data for a week taken across 25,000 premature babies (no sex is reported). The log of the median and its rate of change are used for clarity. **(A)** Graph reflects the actual median weight (kilograms) across days 150–350 (top panel) and corresponding generalized coefficient of variation (CV). **(B)** Graph reflects the daily rate of change in the median weight across days 150–350 (top) panel and corresponding generalized CV bottom panel enclosed in the box. Stars mark inflexion points according to the generalized CV.

The shift into high signal content and statistical regularities conducive of a predictable and controllable neural code was absent from the fluctuations in motor performance of these HR babies. Furthermore, their AIMS scores were significantly lower than those in the TD group. Importantly, the stunting in neuromotor control development was detectable as early as the first visit in the study and persisted 4 months later. This finding is particularly relevant as clinical scoring systems: (1) rely on visual identification of problems, and thus must wait until signs are visible to the naked eye of the clinician administering the inventory, and (2) are sporadically administered. In contrast, the indexes derived from the wearable sensing data can begin to be obtained right after birth and continuously monitored during the first months of life when the baby changes at an accelerated rate (e.g., Figure A1 for typical and Figure A2 for preterm cases).

From these results, we propose to use the uncovered relationship between the rates of change in physical and neuromotor control growth as an index of neurodevelopment so as to detect risk of stunting in neonates. Specifically, typical neurodevelopment should manifest in linear congruence with high *R*^2^-value between the rates of change of physical and neuromotor control growth. Absence of this relation should flag stunting in neonatal neurodevelopment.

### Implications for Mobile Health and Precision Medicine in Pediatrics

The present results have implications for m-Health in the areas of neonatal care. The advent of wearable wireless sensing technology calls for new analytics that enable personalized assessment and continuous monitoring. Neonatal care is one area where it will be possible to utilize objective and automated assessments of the kinds presented here, thus opening the possibility of transferring the daily monitoring from the clinical settings into the home environment. This will be a transformative step in clinical and research areas that will enable uncovering, at a very early stage of life, critical aspects of the non-linear, highly dynamic and stochastic features of neurodevelopment. Following initial screening, this technology may enable easy remote monitoring of infants who are perceived as at risk of neuromotor developmental delay. Through this process – combining wearable sensing technology with bespoke analytics to process longitudinal, yet non-linear data – parents may provide pediatricians and health-care providers with rich continuous information to guide informed medical advice and decisions, rather than relying on relatively short and sporadic clinical visits. Indeed, the marked increase in the prevalence of neurodevelopmental disorders worldwide calls for such transformative changes in the interactions between parents and clinicians as well as those between clinicians and researchers.

### Implications for Neurodevelopmental Research

The analytical methods presented in this paper enable examination of neurodevelopment through the lens of the classical kinesthetic reafference principle, thus connecting in closed loop the neurodevelopment of the PNS and the CNS. This principle states that “*Voluntary movements show themselves to be dependent on the returning stream of afference which they themselves cause*” (20). By examining the baby’s self-produced bodily (leg) motions at the periphery and longitudinally monitoring the transitions from spontaneous random noise to well-structured signals, we were able to characterize the initial stages of acquiring (or not, as the case may be in HR infants) central voluntary control. This is the first time that we can characterize such transitions using an index that depends on the evolution of both physical and neuromotor control growth. In this sense, the present work opens the possibility of drafting a new type of (dynamic) growth chart showing, not only the curves of incremental physical growth but also showing the curves of incremental neuromotor control growth along with the correspondence index between the two. Deviations from the coupled linear relation uncovered by this work could thus flag risk of stunting along either one or both of these (objective) physical parameters. Furthermore, using subjective clinical inventories of functional milestones (e.g., the AIMS, among others) can help complement these newly proposed dynamic charts and provide a more complete profiling of the fast-growing and fast-developing newborn infant.

Looking ahead in neurodevelopment, we may want to consider the infant’s brain transitioning into voluntary (volitional) control over the physical body as a precursor of representational volition and intentional thoughts reflecting decisions to be made based on estimated sensory consequences from impending actions. This form of prospective motor control would lead to deliberate autonomy of the brain over the body, a key element scaffolding the emergence of timely cognitive representations of the body and its surroundings. In this sense, the present results concerning the quantification of noise transitioning to signal, and the failure to do so, could also flag risk of slowing down the development of important foundational components of cognitive and social axes. Here, we posit that sensory reafference – whether arising internally from self-generated movements or externally from other senses – ought to be considered as a key ingredient for the development of cognitive and social abilities.

Disruption in the evolution and maturation of motor output, taken as a form of kinesthetic reafference in the newborn, should therefore raise a flag for risk of neurodevelopmental stunting concerning volitional control. This in turn should alert us of potentially negative future consequences hindering the emergence of intentional cognition. Here, we characterize the lack of neurodevelopmental maturation in terms of noise-to-signal transitions. We propose that such principles should be systematically researched and validated to permit incorporation of the present indexes into early clinical criteria for neurodevelopmental disorders at large.

### Data-Driven vs. Clinically Informed Approaches

The assessments performed in the present work were of two kinds. In one case, we used the clinical labels that the data came with originally to examine the groups of babies accordingly. In the other case, we performed the analyses without pre-classifying according to clinical labels and rather let the variability inherently present in the data reveal self-emerging groups of babies.

The case where we used clinical labels was informative as it helped us gain a general sense of the potential differences that may be detectable already by observation by the clinician. Here, our analysis confirmed that babies born with such complications did indeed differ on average (with marked statistical significance) from typically born babies. Interestingly, this was particularly so when the data were examined through an incremental, first-derivative lens. However, when absolute scores were used these differences were missed. These results underscored the importance of considering the incrementally changing data reflecting the dynamic and non-linear nature of neonatal development.

In the data-driven approach case, we first examined the data without grouping by clinical labels and only afterward compared the outcome of data-driven analyses to the clinical labels. Using the data variability we let stochastic patterns self-emerge then *a posteriori*, we were able to see within each self-emergent group which babies of the self-emergent clusters had been typically born vs. born with complications. Notably, this approach revealed that two of the babies that fell in the statistically TD data-driven group (i.e., with congruent physical and neuromotor growth) had in fact been born with complications and thus clinically defined as such. These babies had received intensive physical therapy. This result confirmed the importance of early intervention when the nervous system is rapidly changing and very likely at its highest degree of plasticity. Likewise, we identified cases where individual babies were clinically labeled as typically born, and thus at no risk, yet were in fact lagging behind in their physical and neuromotor control rates of growth. This again confirms the variable nature of neurodevelopment and illustrates the importance of identifying a type of risk that no parent would want to miss. Indeed, many babies that are typically born go on to receive a diagnosis involving at least one neurodevelopmental disorder later, generally after 3 years of age.

In summary, even though the data from the babies came with clinical labels, by examining their patterns while using data-driven strategies, we were able to automatically identify cases of babies that were labeled as clinically at no risk of developmental delay and yet stunted vs. cases of babies that were labeled at risk from birth complications and yet recovered and went on to form part of the self-emerging data-driven TD group. By examining the data without assigning *a priori* clinical labels and then comparing it *a posteriori* with the clinical labels, we were able to identify patterns that we would have missed had we exclusively relied on the *a priori* clinically labeled data. These overall results underscore the importance of incorporating data-driven approaches in clinical settings in general but in particular, in neonatal Pediatrics.

### Limitations of the Present Study

The present work has several limitations. One is the relatively low number of infants (36) we had access to for the longitudinal assessment. The other is the frequency of the assessments. Despite high power for the estimation process (each sensory–motor estimate includes thousands of data measurements for each individual), and the fact that under this framework each individual is its own control; it would have been ideal to have access to a larger number of infants to track longitudinally. Under such conditions, it may have been possible to uncover self-emerging patterns across various populations of premature infants and infants born normally, within ideal conditions. Moreover, the present study tracked each infant across a span of 6 months, measuring growth and fluctuations in motor performance every 2 months. The first visit ranged from 8 months in typical group or 1–18 months in the group CAR. Based on the results that we quantified in the first visit (Figure 3C), we believe that if these measurements had been performed every day for the first month of life, we may have been able to uncover signs of neurodevelopmental stunting even earlier.

We used the waveforms output by temperature and motion sensors because they were accessible to us in non-intrusive ways. However, it would have been ideal to use them in combination with other rhythms of the newborn’s nervous systems. For example, the same statistical platform that we used here could have been applied to time series of waveforms from respiratory or feeding (sucking) rhythms (11, 12, 26) easy to harness in the NICU or in typically newborn infant wards (27). Feeding and non-feeding sucking patterns require precise motor control from orofacial structures. As such, they can be a precursor of voluntary control that the nervous systems of the newborn may come to self-discover and transfer to the control of bodily rhythms at large. This is a testable hypothesis using the present individualized statistical methods.

Finally, a striking limitation lies in the lack of transparency that we encountered when trying to reproduce the growth charts produced by the WHO (see Appendix). As clearly illustrated within the WHO methodological paper, data used for the formulation of these charts were ultimately obtained, transformed, and smoothed before conforming to the normal distribution. Although we used the rate of change version of these data to be congruent with the skewed distributions of our empirical data (shown in Figure S7 in Supplementary Material), we have no way of recovering the original data reported in such charts. Thus, we utilized the reported Box–Cox power transformation parameter *L* (Figure A1C) to assess the evolution in the skewness of the underlying distributions, and as such the failure of the underlying data to be normally distributed. We also examined the median parameter to estimate growth variables (e.g., weight in Figure A1) and its variability according to the reported generalized coefficient of variation *S*. The latter was not explained with sufficient clarity in the methods searched, and so we are not exactly sure about the lengths of the confidence intervals and/or whether additive or multiplicative statistics were considered in such estimations.

The ability to blindly reproduce published results is a cornerstone principle of basic science, thus the ambiguity surrounding the production of such charts is concerning – particularly given the core critical use of such clinical charts at present. In this case, though we tried hard, we failed to find the proper ways to reproduce the published data because critical information to that end was not available (see Appendix for details). To build Figure A1, we utilized data available to the public. Notwithstanding these limitations, the message that Figure A1 conveys is clear: refrain from imposing normality and linearity in data that is inherently otherwise.

### Conclusion and General Implications of These Results for Early Intervention Programs

In sum, this work illustrates the importance of preserving and respecting the underlying statistical nature of the data we register and of the non-linear features of the phenomena that we set to study. Enforcing assumptions of normality or linearity on the data to simplify our research task will only deter us from truly understanding and resolving the problem at hand. With the epidemic proportion of neurodevelopmental disorders worldwide, the current practices in basic science and clinical settings may have to change and be subject to more public scrutiny to improve patient care. Indeed, early infancy is a critical time, when the individual grows and develops at a uniquely fast rate – which is currently, overlooked using traditional methods. Through the investigation of standardized growth charts and kinematic data, we have highlighted that neurodevelopmental risk can be detected at a very early stage – a step currently masked by insistence on measuring this highly complex, stochastic non-linear dynamical system, as a simple, deterministic, linear, static one.

## Author Contributions

ET conceived and performed data analyses and wrote the paper. BS designed the experiment, provided the data, and edited the paper. CW and SM performed the data analyses and edited the paper. MB edited the paper.

## Conflict of Interest Statement

ET and Rutgers University hold provisional patents for the methods and data types. No other potential conflict of interest is declared.

## Appendix

Standardized population growth charts have been created and produced by both the Center for Disease Control (5) and the World Health Organization (6). Given discrepancies and variation in data collection and thus chart formulation, clinical guidelines suggest the adoption of the WHO charts (28). These growth charts have been adjusted for breast-feeding, race, sex, and other important parameters that the WHO has considered (28, 29). Since 2006, the standard charts have been adopted by over 140 nations, including the United States (US), and remain the gold standard to assess mortality indexes and determine milestones of physical growth. Although not intended to be used as diagnostic tools, these charts are often used by pediatricians to infer general aspects related to neurodevelopmental progress. Despite the statistical rigor they conform to (30, 31), the use of incremental (velocity-dependent) values to better capture the rates of change of the infant, and the use of a handful of motor milestones to track progress, these charts alone may not necessarily capture the true nature of early neurodevelopment – particularly those aspects of neurodevelopment pertaining to the type of neural control of movements reached by 4–5 years of age, also the limiting age of the growth charts.

### Total Number of Babies/Trait

Reported by the WHO:

*weight*: 26,985 (13,623 girls, 13,362 boys);
*length*: 27,334 (13,783 girls, 13,551 boys);
head *circumference*: 27,339 (13,798 girls, 13,541 boys).

From Table A1: number of observations used in construction of WHO child growth standards.

### Longitudinal/Cross-sectional

**Head circumference for boys and girls**: longitudinal samples used until 24 months; start incorporating cross-sectional at around 18–20 months.

**Length for boys and girls**: longitudinal samples used until 24 months; start incorporating cross-sectional at around 18–20 months.

**Weight for boys and girls**: longitudinal samples used until 24 months; start incorporating cross-sectional at around 18–20 months.

### Quoted Methods

The methods from (30) are quoted here given their critical importance for reproduction of results and the hurdles that we encountered in our attempt to understand why/how the data were enforced to be normally distributed.

To avoid the influence of unhealthy weights for length/height, observations failing above +3 SD and below -3SD of the sample median were excluded prior to constructing the standards … The data were split into two sets: one set with all points above the median and another with all points below the median. For each of the two sets, mirror values were generated to create symmetrically distributed values around the median for the upper and lower sets … The distributions of some anthropometric data used in the growth charts are skewed. To remove skewness, a power transformation can be used to stretch one tail of the distribution while the other tail is shrunk. A Box-Cox transformation can make the distribution nearly normal (32). The assumption is that, after the appropriate power transformation, the data are closely approximated by a normal distribution (33). The transformation does not adjust for kurtosis, which is a less important contributor to non-normality than skewness (34). In the LMS technique, three parameters are estimated: the median (*M*), the generalized coefficient of variation (*S*), and the power in the Box-Cox transformation (*L*). The *L* reflects the degree of skewness. The LMS transformation equation is:

$$X=M{\left(1+\text{LSZ}\right)}^{1/L}L\neq 0$$

$$X=M\;{\text{e}}^{\left(\text{SZ}\right)}L=0$$

where *X* is the physical measurement and *Z* is the *z*-score that corresponds to the percentile. The key task of the transformation was to estimate parameters *L, M*, and *S*. With estimates of *L, M*, and *S*, values of *X* are connected to the values of *Z* through the above equation. The percentile is obtained from a normal distribution table where the *z*-score corresponds to the percentile of interest. For example, a *z*-score of 0.2019 corresponds to the 58th percentile. In the case of growth charts, with the *L, M*, and *S* parameters, it is possible to evaluate any single measure in a population as an exact *z*-score or percentile.

Unfortunately, since the original underlying distributions are skewed and the distributions of the rates of change of the physical growth parameters are also skewed, it is not clear how to interpret the generalized coefficient of variation *S* above, derived from the skewed-to-normal transformed data; or even how *S* was obtained in the first place given the different options for additive and multiplicative cases (35–37). This information is critical to obtain an estimate of the growth parameters and their summary statistics. For example, we can gain some sense on the evolution of skewness by looking into the reported *L* parameter (Figure A1C). Yet, we do not know how kurtosis of the underlying probability distributions may change with age and development, given that the power transformation to enforce normality on the data does no adjust for kurtosis. Likewise, the inherent variability to the original data is lost when applying such transformations.

It is not that the statistical methods are flawed. They are sound *under the assumptions made*. The problem rather lies in the assumptions made, as they are not empirically justified. They go entirely orthogonal to the true nature of the empirical data at hand. As such, they erase the very information that we need in order to detect risk for neurodevelopmental derail in the very early stages of life. The information we are searching for is right in front of our eyes, but we are looking at it through the wrong lenses.
