# Supplementary material for: Neonatal Diagnostics: Toward Dynamic Growth Charts of Neuromotor Control
Source: Front Pediatr. 2016 Nov 23;4:121. doi: 10.3389/fped.2016.00121 (PMC5120129; doi:10.3389/fped.2016.00121)
Supplement: Supplementary file 1 [file Table_1.DOCX]

**Supplementary Tables**

**Supplementary Table 1 – Results ensemble stats (temperature dependent motion data)**

| CT | **Source** | **SS** | **df** | **MS** | **Chi-sq** | **Prob>Chi-sq** |
| --- | --- | --- | --- | --- | --- | --- |
|  | Columns | 400.22 | 2 | 200.111 | 6.37 | 0.04 |
|  | Error | 1233.78 | 36 (3 visits) | 51.407 |  |  |
|  | Total | 1634 | 34 |  |  |  |
| CAR |  | | | | | |
|  | Columns | 24.97 | 2 | 12.487 | 0.09 | 0.9556 |
|  | Error | 15387.03 | 70 (22x3 visits and 2x2visits) | 284.945 |  |  |
|  | Total | 15412 | 68 |  |  |  |

**Supplementary Table 2 – Results ensemble stats (growth data)**

| **Parameter (absolute value)** | **Mean and Variance (Normal PDF)**  **[mean, var]** | **Comparison Wilcoxon rank sum test (p-value)** |
| --- | --- | --- |
| Weight (Kg) | CAR [7.71225, 4.57677]  CT [7.71417, 2.60401] | 0.4231 |
| Head Circumference (cm) | CAR [42.8155, 9.9399]  CT [43.5032, 12.234] | 0.2750 |
| Body length (cm) | CAR [67.3408, 44.2836]  CT [66.7217, 30.1483] | 0.4681 |
| **Parameter (Δ since birth)** | **Mean and Variance (Lognormal PDF)**  **[mean var]** | **Comparison Wilcoxon rank sum test (p-value)** |
| Weight (Kg/day) | CAR [0.0309341, 0.0001137]  CT [0.0448418, 0.0003427] | 4.5270e-06 |
| Head Circumference (cm/day) | CAR [0.182559, 0.00595036]  CT [0.274164, 0.0238794] | 7.1014e-04 |
| Body length (cm/day) | CAR [0.284353, 0.0130271]  CT [0.418218, 0.0531367] | 0.0015 |
| **AIMS scores**  **(absolute values)** | **Mean and Variance (Normal PDF)**  **[mean, var]** | **Comparison Wilcoxon rank sum test (p-value)** |
| Total | CAR [23.2817, 214.09]  CT [29.2778, 206.206] | 0.0288 |
| Prone | CAR [8.30986, 42.9883]  CT [11.5556, 35.9111] | 0.0074 |
| Supine | CAR [6.38028, 5.66761]  CT [7.38889, 4.24444] | 0.0341 |
| Sitting | CAR [5.42254, 15.1618]  CT [6.61111, 18.073] | 0.1883 |
| Standing | CAR [3.16901, 8.9996]  CT [3.72222, 9.40635] | 0.1187 |
| **AIMS scores**  **(rate of change/day since birth)** | **Mean and Variance (Lognormal PDF)**  **[mean, var]** | **Comparison Wilcoxon rank sum test (p-value)** |
| Total | CAR [0.082312, 0.00137389]  CT [0.140831, 0.00089313] | 1.1916e-11 |
| Prone | CAR [0.0295296, 0.000435786]  CT [0.0541267, 0.000169476] | 1.6701e-10 |
| Supine | CAR [0.0246442, 8.12808e-05]  CT [0.0409908, 0.00020606] | 1.5132e-09 |
| Sitting | CAR [0.0183238, 0.000123955]  CT [0.0318401, 0.000152011] | 4.1693e-05 |
| Standing | CAR [0.0110414, 4.0511e-05]  CT [0.0174758, 8.00733e-05] | 9.8285e-06 |

**Supplementary Table 3: Estimated parameter ranges**

Blue entries are for the babies in the data-driven classification of the typically developing (TD) group. This is the group formed by babies with congruence in the rate of change in physical and in neuromotor control growth (Main text **Figure 5** A-C G1 group with 0.89, 0.89, 0.83 for adjusted R^2^ fit for Δweight/day, Δbody-length/day and Δhead circunf/day. Note that two clinically labeled babies born with complications made it to this group. Their rates were congruent albeit they were the last in this ranking. Gray entries are the babies corresponding to the data-driven classification of G2. This group is a mixture of babies born with and without complications. The red entries are the data-driven classification of babies at high risk of stunting in neurodevelopment. Only one baby from the clinically labeled CT group (born without complications) made it into this G3 cluster. The rest were babies born with complications.

| Physical Growth Ranking | ΔPhysical growth,  Δweight, Δbody-length,  Δhead circunf, ΔAIMS | Ranges Estimated  µ, σ^2^, sk, kt from Norm MaxAcc | Mean Temperature / visit |
| --- | --- | --- | --- |
| CT02 | 0.0336, 0.3067, 0.2011, 0.1204 | 0.4922 0.6029;0.0000 0.0151;  -5.8394 5.5892;1.4933 37.1442 | 33 31 31 |
| CT05 | 0.0306, 0.3369, 0.2213, 0.0655 | 0.4979 0.5912; 0.0000 0.0170;  -5.1801 6.0122;1.5303; 38.3716 | 32 30 32 |
| CT09 | 0.0211, 0.2067, 0.1259, 0.0497 | 0.4920 0.58 2; 0.0000 0.0139;  -5.2404 5.9818;1.5586 38.1043 | 30 27 31 |
| CT10 | 0.0304, 0.3003, 0.1998, 0.0545 | 0.4806 0.6550; 0.0000 0.0203;  -5.0611 5.9364;1.5428 36.5140 | 32 28 30 |
| CT11 | 0.0306, 0.2268, 0.1369, 0.0449 | 0.4950 0.5706;0.0000 0.0156  -5.3382 6.1202;1.6040 38.6636 | 26 29 30 |
| CT12 | 0.0388, 0.3465, 0.2308, 0.1081 | 0.4805 0.6040;0.0000 0.0241  -5.1968 5.8017;1.3588 42.2956 | 24 29 27 |
| AR11 | 0.0249, 0.2426, 0.1545, 0.0899 | 0.4952 0.5879;0.0000 0.0175  -5.1872 6.2760;1.5233 42.5557 | 28 29 31 |
| AR26 | 0.0423, 0.4470, 0.2651, 0.1233 | 0.4937 0.6062;0.0000 0.0166  -4.6174 5.4958;1.3735 32.7174 | 27 28 29 |
| CT01 | 0.0513, 0.4684, 0.3146 0.1494 | 0.4966 0.6152;0.0000 0.0214  -5.0672 5.7469;1.5407 35.9470 | 31 32 32 |
| CT03 | 0.0420, 0.3145, 0.2146 0.1455 | 0.4914 0.5746;0.0000 0.0129  -4.8887 5.8321;1.3109 35.0186 | 31 31 31 |
| CT06 | 0.0232, 0.1730, 0.1133, 0.1256 | 0.4957 0.5925;0.0000 0.0261  -5.2723 5.9838;1.5056 37.2169 | 31 32 31 |
| CT08 | 0.0514, 0.4573, 0.2895 0.1305 | 0.4964 0.5814;0.0000 0.0141;  -5.6104 6.8170;1.3318 51.2641 | 31 31 31 |
| CT04 | 0.0247, 0.2561, 0.1682, 0.0514 | 0.4875 0.5647;0.0000 0.0082  -5.0714 5.9913;1.3845 42.1141 | 26 25 29 |
| AR24 | 0.0349, 0.3925, 0.2757 0.0562 | 0.4960 0.5640;0.0000 0.0084  -4.7638 5.9219;1.5891 40.6918 | 30 33 33 |
| AR10 | 0.0370, 0.3510, 0.2318 0.0614 | 0.4960 0.5640;0.0000 0.0084  -4.7638 5.9219;1.5891 40.6918 | 30 33 33 |
| AR12 | 0.0227, 0.1899, 0.1250, 0.0490 | 0.4960 0.5640;0.0000 0.0084  -4.7638 5.9219;1.5891 40.6918 | 30 33 33 |
| AR20 | 0.0597, 0.3566, 0.2530, 0.0389 | 0.4960 0.5640;0.0000 0.0084  -4.7638 5.9219;1.5891 40.6918 | 30 33 33 |
| CT07 | 0.0330, 0.2791, 0.1923, 0.1672 | 0.4875 0.5647;0.000 0.0082; -5.0714 5.9913;1.3845 42.1141 | 26 25 29 |
| AR02 | 0.0878, 1.0269, 0.6729, 0.1512 | 0.4918 0.5887;0.000 0.0133;  -4.5870 5.9431;1.4500 37.1913 | 29 29 0 |
| AR07 | 0.0326, 0.2483,0.1579, 0.1622 | 0.4875 0.5647;0.000 0.0082;  -5.0714 5.9913;1.3845 42.1141 | 26 25 29 |
| AR15 | 0.0305, 0.2402, 0.1509, 0.1306 | 0.4897 0.5865;0.0000 0.0162  -5.7218 5.9192;1.4135 37.1985 | 28 29 31 |
| AR22 | 0.0622, 0.6502, 0.4481, 0.1422 | 0.4944 0.5818;0.0000 0.0151  -4.4427 5.4823;1.5967 34.3096 | 30 31 31 |
| AR03 (2V) | 0.0375, 0.4480, 0.2854, 0.1021 | 0.4927 0.5816;0.000 0.0142;  -6.0128 8.0853;1.3809 68.2065 | 29 31 na |
| AR08 (2V) | 0.0310, 0.2408, 0.1518, 0.1197 | 0.4966 0.5772;0.0000 0.0092  -5.2981 5.5263;1.5935 31.7060 | 30 30 na |
| AR04 | 0.0322, 0.2826, 0.1842, 0.1244 | 0.4944 0.5818;0.0000 0.0151  -5.8163 6.0338;1.5967 38.4325 | 29 31 31 |
| AR05 | 0.0717, 0.6632, 0.4226, 0.1729 | 0.4951 0.6126,0.000 0.0182;  -5.1327 7.4416,1.4944 62.6376 | 33 32 30 |
| AR06 | 0.0241 0.1503 0.0884 0.0573 | 0.4935 0.5884,0.00 0.0127;  -5.6375 5.9706;1.0000 37.4963 | 31 31 33 |
| AR09 | 0.0238 0.1622 0.1001 0.1028 | 0.4914 0.5746;0.00 0.0129;  -4.8887 5.8321;1.3109 35.0186 | 31 31 31 |
| AR13 | 0.0267 0.2162 0.1395 0.1237 | 0.4930 0.5808;0.00 0.0114;  -5.5550 7.9772;1.5066 67.8095 | 33 31 31 |
| AR14 | 0.0218 0.2004 0.1266 0.1283 | 0.4887 0.5678;0.00 0.0127;  -5.4733 5.6923;1.4947 40.4251 | 33 29 32 |
| AR16 | 0.0191 0.1817 0.1185 0.1111 | 0.4936 0.5793;0.00 0.0107;  -5.3564 6.4949;1.4653 43.8045 | 30 33 30 |
| AR17 | 0.0196 0.1969 0.1325 0.0531 | 0.4961 0.5874;0.00 0.0112;  -6.8307 7.1600;1.5137 54.6354 | 31 33 35 |
| AR18 | 0.0257 0.2216 0.1396 0.1258 | 0.4945 0.5593;0.00 0.0072;  -4.9323 6.1556;1.5738 39.2848 | 30 30 31 |
| AR19 | 0.0297 0.4415 0.2795 0.0777 | 0.4964 0.5814;0.00 0.0141  -5.6104 6.8170;1.3318 51.2641 | 31 31 31 |
| AR21 | 0.0333 0.2957 0.1761 0.0711 | 0.4960 0.5640;0.00 0.0084;  -4.7638 5.9219;1.5891 40.6918 | 30 33 33 |
| A25 | 0.0249 0.2426 0.1545 0.0899 | 0.4896 0.6193;0.00 0.0204;  -5.2763 5.46781.5287 32.1816 | 26 30 24 |
