# Supplementary material for: Neonatal Diagnostics: Toward Dynamic Growth Charts of Neuromotor Control
Source: Front Pediatr. 2016 Nov 23;4:121. doi: 10.3389/fped.2016.00121 (PMC5120129; doi:10.3389/fped.2016.00121)
Supplement: Supplementary file 2 [file Data_Sheet_1.pdf]

Torres et al
Supplementary Material

New Data Type Integrates Autonomic and Sensory-Motor signals

We define a new data type that integrates fluctuations in temperature readings with fluctuations in motor performance. This bundle of signals related to the autonomic and sensory-motor systems, allows us to better explore and identify motion signatures that have the potential to be physiologically relevant to both the peripheral and central nervous systems (PNS and CNS).

The temperature and the motion (acceleration and gyroscopic) signals are simultaneously registered at the same sampling resolution, 128Hz. The sensors output temperature readings related to several temperature types: skin surface temperature, ambient temperature and temperature related to the battery's energy consumption indicative of the amount of actively generated motion output. The higher the amount of actively generated movements, the more battery energy is drawn, and thus the higher the temperature is (on average). Yet, because there is also adaptation between the person's skin surface and the sensor's surface, the temperature readings fluctuate. Importantly, temperature readings have been found to fluctuate differently if the person is actively *vs*. passively moving. Specifically, slow rates of change in temperature fluctuations correspond to passively generated motions (as when the person is moved and not generating large motions.) In contrast, actively self-generated motions, particularly those which are larger and more systematic, result in higher rates of change in temperature fluctuations.

These temperature fluctuations can therefore be captured and analyzed to provide an indication as to of the amount of adaptive motion from self-generated movements that the individual

wearing the sensors performs. Sample temperature fluctuations for one sensor are shown in **Supplementary Figure 1B** – these data are from continuously registered motions for over 8 hours. This figure illustrates the raw data along with the smoothed data, highlighting the maxima and minima of the waveform (smoothing achieved using standard MATLAB signal processing software, MATLAB Release 2014b, The MathWorks, Inc., Natick, Massachusetts, US.)

Given a range of temperature, it is possible to isolate all the motion that takes place within that range because the signals from both instruments are synchronously registered at the same sampling resolution. For example, red dots in **Supplementary Figure 1B** mark all the temperature registered for movements between 15.5-15.6 m/s^2. (These are arbitrarily selected for illustration purposes only.) **Supplementary Figure 1C** shows the acceleration computed from the triaxial traces, i.e. the magnitude of the acceleration vector giving the scalar quantity,

$$acc = \sqrt{acc_x^2 + acc_y^2 + acc_z^2} \qquad (1)$$

The terms in (Equation 1) are the components of the tri-dimensional acceleration vector.

Given the motion registered in some time period, it is also possible to determine movement segments occurring within a specific temperature range. For instance, the acceleration trace in **Supplementary Figure 1C** shows segments highlighted for specific temperature intervals between 28-29°C (red) and 25-26°C (yellow). (These are arbitrarily selected for illustration purposes only.)

Supplementary Figure 2 shows the matrices of motion expressed as a function of temperature, allowing the selection of all the acceleration that takes place for each sensor for each 2°C-interval. **Supplementary Figure 2B** shows the color maps of these data in minute-by-minute

matrices across a complete duration of 750 minutes, demonstrating the maximal deviations from the mean acceleration for each of the sensors. The color bar highlights the range of m/s^2 values. **Supplementary Figure 2C** breaks down the ranges of maximal deviation from the mean acceleration as time-series of the peaks in the order in which they were registered over the time period of 750 minutes for each 2°C-interval (color-coded in ascending order) for the right sensor. This panel also shows the range of temperatures in the color bar. For each of these acceleration regimes per temperature interval we obtain the peaks to analyze the fluctuations in amplitude and timing. This paper focuses on the stochastic analyses of fluctuations in amplitude using a new statistical platform for the personalized analyses of natural behaviors.

Statistical Platform for the Personalized Analyses of Natural Behaviors

The overall distribution of peaks across the data set is obtained. The mean value is empirically estimated and used as reference to determine the maximal deviations from it. The time-series of these fluctuations in maximal deviations from the empirically estimated mean provides the waveform of interest for our analyses. These are spike trains of random fluctuations in amplitude. Note here that the temporal order of the peaks is retained but the actual timings do not enter in the present analyses. The values of the fluctuations in amplitude of the waveform are normalized (Mosimann 1970) to avoid possible allometry effects due to differences in the sizes of the limbs of the babies (Lleonart, Salat et al. 2000). These normalized fluctuations define the micro-movements of the waveform.

For each segment between two minima, we obtain the normalized peak acceleration (*NPeakAcc*):

$$NPeakAcc_{\text{min to min}} = \frac{PeakAcc_{\text{min to min}}}{PeakAcc_{\text{min to min}} + Avrg\left(Acc_{\text{min to min}}\right)} \qquad (2)$$

Larger values of this index indicate slower acceleration on average, since smaller averaged acceleration values in the denominator result in higher values of the index. These values across the same number of hours of recording, for each visit and each baby, are then gathered into a frequency histogram using optimal binning algorithms (Freedman and Diaconis 1981, Shimazaki and Shinomoto 2007).

For each minute block of each hour (1,200 readings from 20Hz x 60 seconds) in the raw data time-series we extract the peaks as described above and normalized them. These new series of normalized peaks (retaining the order in which they were acquired) are treated as spike trains representing a continuous random process under identically independent distributed (i.i.d.) assumption. More specifically they are input to a continuous Gamma process. To this end we use maximum likelihood estimation (MLE) to estimate the shape and scale parameters of the continuous Gamma family of probability distributions. We sweep through the original time series and estimate the Gamma-parameters for every minute-block to construct a continuous stochastic trajectory. We use the same number of hours for all babies (i.e. 8 hours).

The points of the estimated trajectory are plotted on the Gamma parameter plane. Each point on this plane represents a probability distribution with estimated shape and scale parameter. These steps are illustrated in **Supplementary Figure 3A.** The first panel of this figure shows sample frequency histograms from typical (left) and atypical (right) data sets. The third panel shows sample estimated Gamma probability density functions (PDFs). The fourth panel represents the Gamma parameter plane with the two estimated points from the frequency histograms. The estimated shape and scale parameters are plotted with 95% confidence intervals for each estimated value. The last panel of **Supplementary Figure 3A** shows sample data points estimated in successive steps to illustrate their shifts over time, thus reflecting the non-stationary

feature of the data. The shifts occur at different rates. The stochastic trajectories these shifts give rise to are the center of our analyses (see below) as they track the changes in dispersion and shape of the distributions.

To track the evolution of the noise-to-signal transitions, the Gamma parameter plane is further divided into four quadrants using the medians of the family of estimated shape and scale parameters each day. **Supplementary Figure 3B** illustrates this step. The vertical line is the median across all estimated shape values. The horizontal line is the median across all estimated scale values. Each point is color coded according to the temperature value of the motion (color bar displays the full range for each visit.)

We next plot the PDFs in **Supplementary Figure 3C**. Their color represents the temperature value as in **3B**. Further since in the case of the Gamma distribution function, the scale value is also the noise-to-signal ratio (a.k.a. the Fano Factor (Fano 1947)), we track the fluctuations of this value over time:

$$FF = \frac{\hat{\sigma}_w^2}{\hat{\mu}_w} \qquad (3)$$

In (Equation 3) the numerator denotes the estimated variance and the denominator the estimate mean for the window of data in the time series. This noise-to-signal ratio is a measure of dispersion. The relation between the FF and the estimated scale Gamma parameter *b* is shown in equation (4):

$$b = \frac{\hat{\sigma}_w^2}{\hat{\mu}_w} = \frac{\left(a.b^2\right)_w}{\left(a.b\right)_w} \qquad (4)$$

Tracking the rates of change of adaptive neuromotor control: Noise-to-signal transitions on the Gamma parameter plane

The Gamma parameters shift positions on the Gamma plane as we re-estimate them minute-by-minute. We track the non-stationary noise-to-signal ratio (FF) within a day over the course of 8 hours as the values transition between the left-upper quadrant (LUQ) and the Right Lower Quadrant (RLQ) of the Gamma parameter plane partitioned by the median values of the estimated shape and scale parameters. We also track these noise-to-signal transitions from visit to visit. We track the *amplitude* and the *frequency* of the transitions (described later).

The RLQ contains the points with the lowest noise-to-signal ratio (lowest values of the estimated Gamma scale b-parameter), while the LUQ contains distributions with higher dispersion. Points in the LUQ have higher noise-to-signal ratio and are characterized by more skewed shapes tending towards the limiting case of the Gamma plane, where the shape parameter has a value 1; this is the case representing the "memoryless" Exponential distribution.

We define the ΔN as the maximal change in noise-to-signal ratio in one visit. This is the transition between quadrants with the largest amplitude. Using the temperature ranges within which the motion was registered, we can further refine ΔN for each temperature interval. This ability to select motions within regimes of high-fluctuations in temperature provides additional information by more than one re-afferent source (acceleration and temperature) from different autonomic and sensory-motor systems (depicted in **Figure 2**). Both types of fluctuations can be revealing of actively, self-generated motions continuously feeding back to the baby's brain. Thus, it is not just motion that is isolated by these methods. Rather, it is motion that has the potential to be physiologically relevant to the nervous system of the newborn. More precisely,

the exchange of noise and signals associated with temperature fluctuations is monitored with an eye for higher regimes of temperature identified with ***actively self-generated*** motoric rhythms. Within those rhythms the frequency and the amplitude of the fluctuations tending to the RLQ and steadily converging to it are of interest in this work. They give us a sense of contrast between spontaneous random noise in the motions and actively generated signal.

Personalized Analyses: Under this approach it is possible to track the stochastic trajectories of each baby in a personalized manner. In the previous method depicted in **Supplementary Figure 3B-C**, we selectively assessed the motions corresponding to a given temperature range (e.g. binned in 2°C intervals). As we previously showed in **Supplementary Figure 1C**, it is also possible to track the motions and identify which temperature regimes the motion belongs to.

To this end, we track the changes in noise-to-signal ratio in the motions using the above-mentioned 20Hz x 60 second, minute-by-minute blocks for each baby. Within each block of motion we then obtain the corresponding temperature range registered by the sensor. An example of this method is shown in **Supplementary Figure 4** for all three visits of one baby. In each case we plot the median lines from the estimated Gamma parameters (as in **Supplementary Figure 3B**), obtained across the scatter of estimated values. The points in the scatter above or below the median values are color-coded by the temperature values for which that motion occurred.

Panels across **Supplementary Figure 4A** show the log-log Gamma plane while those in panel **4B** show the summary statistics in 4-dimensions (mean along the x-axis, variance long the y-axis, skewness along the z-axis and the size of the marker is the kurtosis). The color map provides the range of temperatures for each noise level in the estimated LUQ and RLQ points. In each case, the points from the RLQ below the median scale value and above the median shape

value are plotted in shades of blue. The points corresponding to the LUQ are colored in shades of red, according to the temperature values within which motions were registered. For example if we have 900 peaks in 1200 points registered in one minute, we can extract the temperature value associated with each peak and provide an average reading of the temperature associated with the estimated Gamma parameters (shape, scale)-point on the Gamma parameter plane. This provides a visual on the prevalence of high (or low) temperature gradients on the Gamma plane. This indicates on average the amount of active motions in each of the quadrants.

Panels **4C** and **4D** show them separately with the color bars depicting the corresponding temperature-ranges for each scatter. These figures demonstrate the evolution from visit to visit of the noise-to-signal in each quadrant along with the amount of active motions. Of interest here is how the RLQ is populated with darker shades indicating increasingly actively generated motions with high signal content (low noise to signal ratio) and a prevalence of symmetric distributions (skewness value of 3 is symmetric distribution).

To compute the rate of change in noise for each visit, the estimated Gamma parameters are divided by the number of days since birth and up to that visit (shown on the LUQ of **Supplementary Figure 4A**). This provides a rate of change (incremental) measure of the stochastic signatures. Then the maximal amplitude change in noise-to-signal ratios ΔN is obtained for each visit by numerically differentiating and subtracting the minimum from the maximum across the LUQ and the RLQ. Likewise we obtain the $\Delta(\Delta N)$ which is the change in noise-to-signal transitions across the visit's stochastic trajectory. This is the rate of change of adaptive neuro-motor change of the infant in response to physical body growth from one visit to the next. In each of the computations of **Supplementary Figures 3** and **4** the stochastic

trajectories are obtained as the Gamma parameters move (non-stationary estimates) across the hours of the day.

Noise-to-signal transitions: During the various hours of data registration the stochastic signatures of the motion-temperature signals may be at times non-stationary. As explained above, we track the noise-to-signal transitions between the LUQ and the RLQ. Specifically, we track the ***magnitude*** of the transition as well as the ***frequency*** of those transitions from one quadrant to the other.

To determine the magnitude of the transition between quadrants we compute the norm of the difference between the estimated (shape, scale) vector at time t+1 and that at time t. For all transitions we obtain temporal profile of these magnitude values (in the order in which they were obtained). Examples of these profiles are shown on **Supplementary Figure 5B** for a representative CT baby with stochastic trajectories (blue) in **Supplementary Figure 5A** and for a representative CAR baby (red trajectories).

Maximum magnitude of the shift: To obtain the time series of shifts in Gamma-parameters we compute the Euclidean norm of the vector difference on the Gamma plane (e.g. **Supplementary Figure 5B** shows those magnitudes for the CT baby trajectories). Then the maximum shift amplitude is obtained across all shifts between quadrants. To this end we take the point in RLQ with the minimum scale value and the maximum shape value (i.e. the PDF with minimum noise-to-signal ratio and maximal symmetric shape) and subtract it from the point in LUQ with the maximum scale value and minimum shape value (i.e. the PDF with maximum noise-to-signal ratio and most skewed shape). The magnitude of the resulting vector on the Gamma parameter

plane is then obtained using Euclidean norm. Then the average value across the 3 visits is set as the point of interest for each baby.

To this end, for each baby, we track the changes in noise-to-signal ratio in the motions using the above-mentioned 20Hz x 60 second, minute-by-minute blocks, with the ½-minute sliding window. Using these blocks we obtain, for each measurement, the corresponding average temperature range within which this windowed motion was registered and color the estimated point accordingly. An example of this method is shown in **Supplementary Figure 4** for all three visits of one baby. In each case we plot the median lines from the estimated Gamma parameters (as in **Supplementary Figure 3B**), obtained across the scatter of estimated values. The points in the scatter above or below the median values are color-coded by the temperature range for which those motions occurred.

The two arrays containing such indexes for the LUQ and RLQ are numerically differentiated to count the number of instances when the change was consecutive within one quadrant (i.e. the difference from one shift to another is 1). In contrast differences from one shift to another that are greater than 1 denote instances when the shifting is from one quadrant to another. For example if the indexes array for the points in the LUQ has values [1,2,3,4,6,9,10,11], then the difference array will be [1, 1, 1, 2, 3, 1, 1]. This means that the process remained stationary in the LUQ for three consecutive times, then jumped to the RLQ, jumping 2 instances and then came back to the LUQ and returned to the RLQ where it jumped three instances before it came back to the LUQ two more consecutive times. We gather these instances in a histogram and normalize each bin by the number of occurrences to obtain the distribution of noise-to-signal transitions, and the proportion of times spent in each quadrant of high or low noise along with the proportion of shifts. Then we compute the percentage of stationary and non-stationary transitions for each

baby. For example, **Supplementary Figure 5C** shows the plots of the outcome measure for the two representative babies with the trajectories in **Supplementary Figure 5A** (one CT and one CAR) for the LUQ case. The insets in **Supplementary Figure 5A** show the histograms of the proportion of time in the stationary and non-stationary states with respect to the LUQ for each baby type (as clinically labeled). Notice that in this **Supplementary Figure 5C** example the CAR baby does not transition much in relation to the CT baby in either of the stationary and non-stationary states. We alert the reader that the results will focus on the type of plot shown in **Supplementary Figure 5C** summarizing the outcome from self-emerging (data-driven) clusters of baby types according to the rates of physical growth and evolution in neuro-motor control. However for illustrative purposes of the methods we used the clinically labeled denomination (CT *vs.* CAR.) Since these methods are personalized and longitudinally track neurodevelopment, we can compare for each baby the clinical label given at birth *vs.* the data-driven clustering our objective (dynamic) classification provided later, as the babies grew and developed their nervous systems.

Results from Analyses Using the a Priori Labeled Clinical Data

Ensemble data shows fundamental differences in the rates of change of temperature-dependent fluctuations in motor performance of clinically pre-labeled baby types

The groups pre-labeled CT and CAR yielded significantly different distributions of fluctuations in temperature according to the Kolmogorov test for empirical distributions, right sensor data $p<1.5x10^{-29}$, left sensor data $p<7.4x10^{-29}$. This difference can also be appreciated in the empirical cumulative distribution function (eCDF), the probability distribution function (PDF) and the probability plots of **Supplementary Figure 6A-C** respectively, for the data of the sensors

positioned on both legs. No differences between the signatures of the left and right legs were noted within either group (*p>0.9* rank sum Wilcoxon test)

The ensemble data from the distributional analyses was also examined for the pre-labeled groups. Differences emerged in the ranges of temperatures within which the babies' motions were registered. Specifically, the CT infants had higher values at the highest density than the CAR babies (34°C CT *vs*. 28°C CAR) as can be appreciated in **Figure 6A** center. The summary statistics from the estimated stochastic signature analyses also revealed differences between the two groups. Specifically, the skewness was the moment that maximally separated the two groups (mean comparison $\overline{p} < .04$, based on comparison of the third moment).

Figures 6D, 6E show the stochastic trajectories across babies of each pre-labeled group along with the frequency of changes in parameter values (shifts) between the left upper quadrant (LUQ) and the right lower quadrant (RLQ) of the Gamma parameter plane, as determined by the median values of the estimated shape and scaled Gamma parameters. The CT group displayed a higher frequency of shifts overall. This lower frequency of noise-to-signal transitions in the CAR group prompted further exploration regarding the inner-quadrant transitions (stationary) *vs.* the inter-quadrant transitions (non-stationary). To that end, in a subsequent section we assess the babies individually, without a-priori labels in search of self-emerging patterns.

Incremental rates of physical growth rather than their absolute values reveal dramatic differences between babies born with and without complications

The absolute data reflecting the measurement of physical parameters in each visit was pooled across each group. This yielded frequency histograms that were well fit by the symmetrical normal distribution. This can be appreciated in **Supplementary Figure 7 (middle column**

insets) for each physical growth parameter. No significant differences were found for absolute values between the groups according to the rank sum Wilcoxon test on the medians. This is reported on **Supplementary Table 1**.

In marked contrast to the absolute values recorded each visit, the incremental data across visits yielded frequency histograms that were well fit by the lognormal distribution. Specifically, these distributions were skewed with long tails to the right and high statistically significant differences were found according to the rank sum Wilcoxon test on the medians. This is reported in **Supplementary Table 1** as well. As we did in **Supplementary Figure 6A-C,** the physical growth rate of change data are displayed in **Supplementary Figure 7** using the eCDF, PDF and probability-plots formats.

Lastly **Supplementary Table 2** reports the AIMS scores and the statistical comparisons for absolute values and incremental data. The former had mixed results with some scores showing statistical differences and others not showing the differences between the two groups. In contrast the incremental AIMS data were more revealing of the differences (as in the rate of growth for each group under examination). These differences were all highly significant when using the rates of change since birth, rather than the actual values registered in each of the visits.

These results on the rates of change of physical growth, motor readiness scores along with those from the previous section involving the differences in fluctuations in temperature and motor performance, prompted us to examine the cohort without a priori clinical labels in search for self-emerging rather than assumed groups.

Supplementary Figures

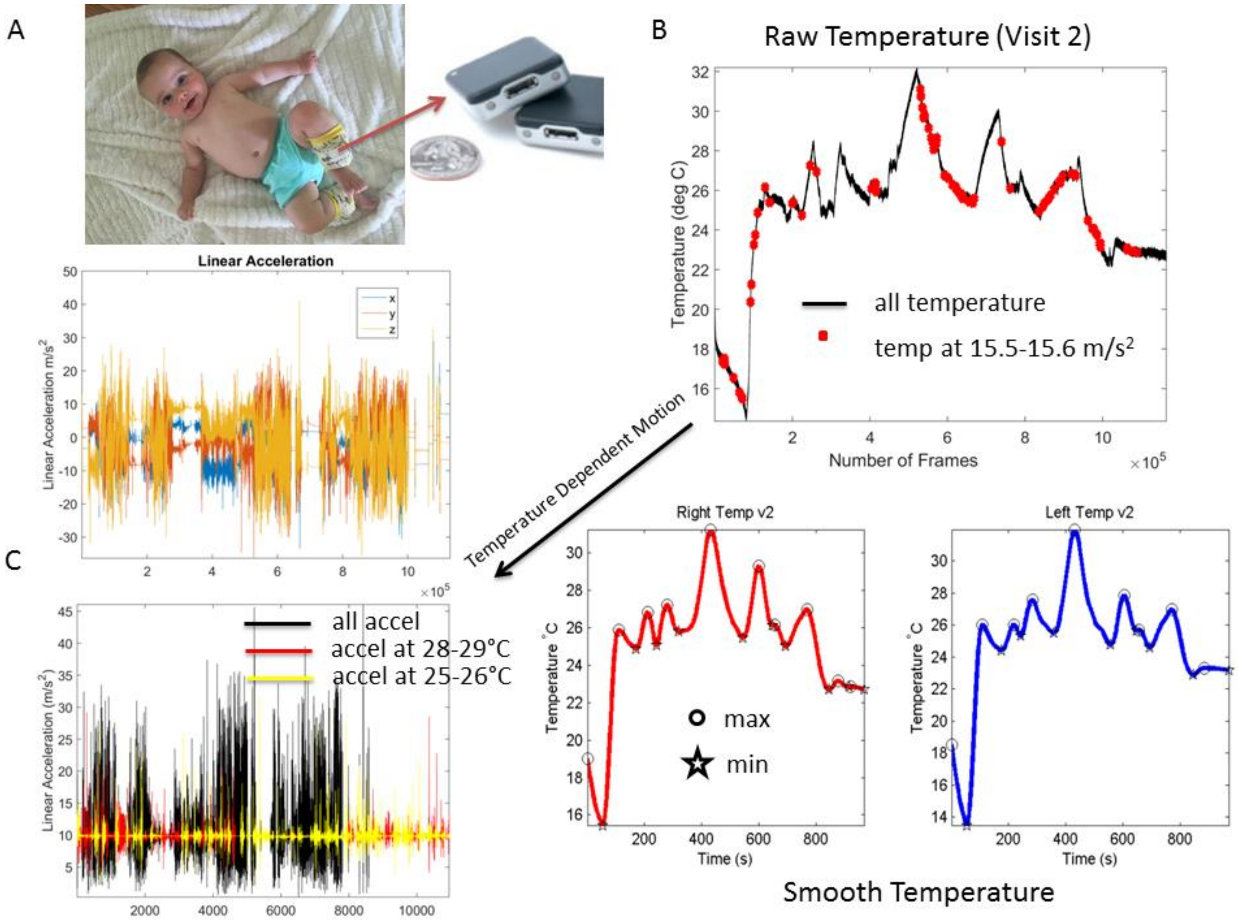

Supplementary Figure 1

Methods of data registration. (A) Inertial measurement units (IMUs) containing accelerometers, gyroscopes and temperature sensors were used to register several physiologically relevant signals partly output by the nervous systems of the baby. These included linear acceleration, orientation and temperature. Sensors (APDM Portland, OR) are light weight (20 g see inset) and record at up to 128HZ (set to 20Hz in these data sets). Sensors were attached to the baby's ankles and recorded for up to 13 hours. (B) Raw temperature traces for one of the visits (randomly chosen) with red landmarks of occurrences of acceleration reaching between 15.5-15.6 m/s^2 (number randomly chosen for explanation purposes only). Traces of the smooth

temperature from the right and left sensors are shown with highlighted local minima and maxima. (C) Linear acceleration traces from the x, y, z directional components and linear acceleration traces of the magnitude built using Euclidean norm. Traces in yellow are acceleration ranges occurring for the 25-26°C-interval, while those in red are occurring at 28-29°C-interval (intervals randomly chosen within the temperature range to illustrate the notion of temperature-dependent motion).

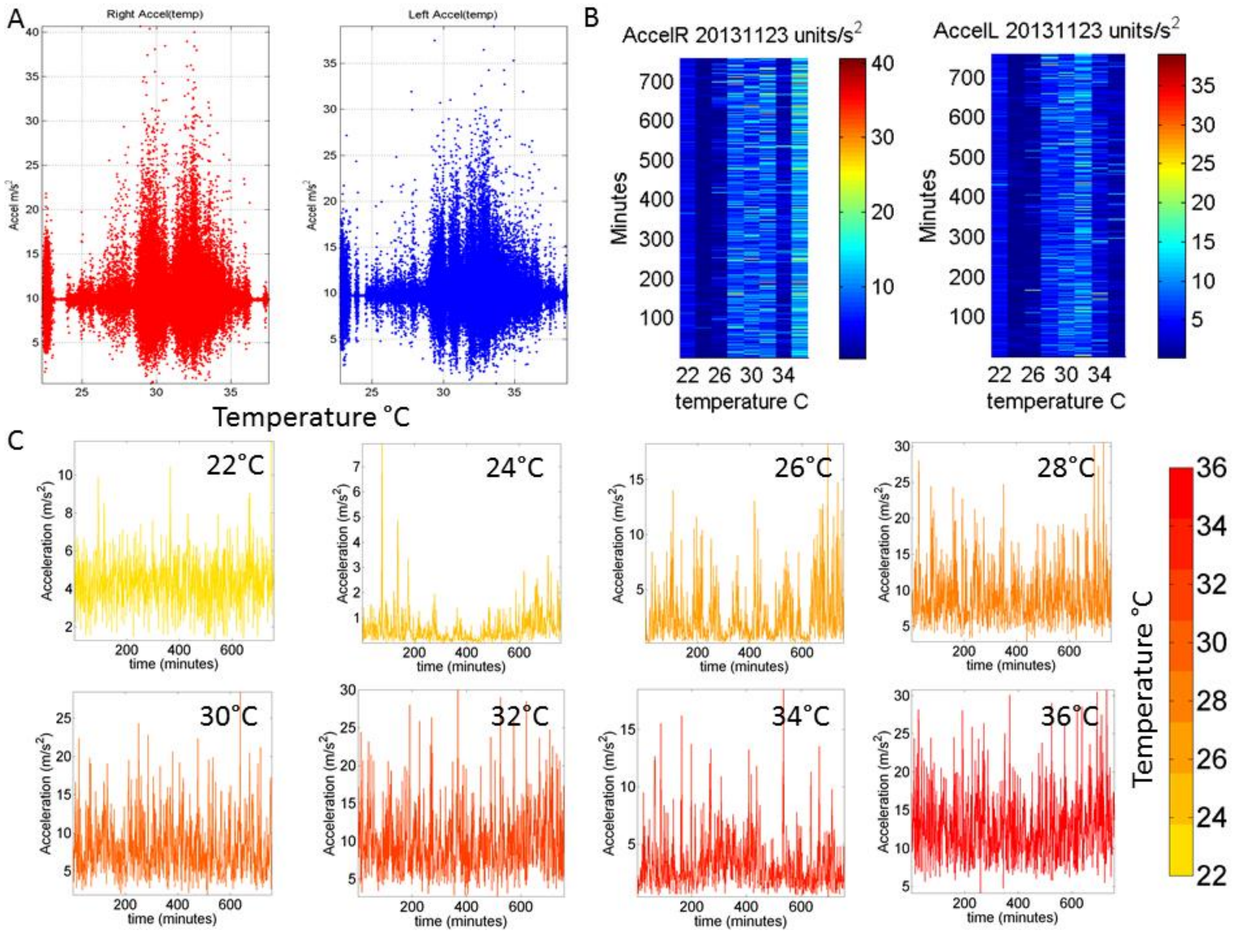

Supplementary Figure 2

Methods to extract temperature-dependent motion. (A) Matrices for the two sensors gathering all the acceleration data registered for each temperature degree. (B) Color map data matrices obtained from the maximal deviation from the mean acceleration in (A) in the time they

were registered at each minute and temperature degree interval. Each interval in this case spans 2°C and the color bar indicates the acceleration range for the maximal deviations from the mean acceleration (m/s^2) for each sensor. (C) Each level of maximal deviation from the mean acceleration per temperature interval is displayed color coded according to the color bar for the number of minutes registered (750 min, 12.5 hours).

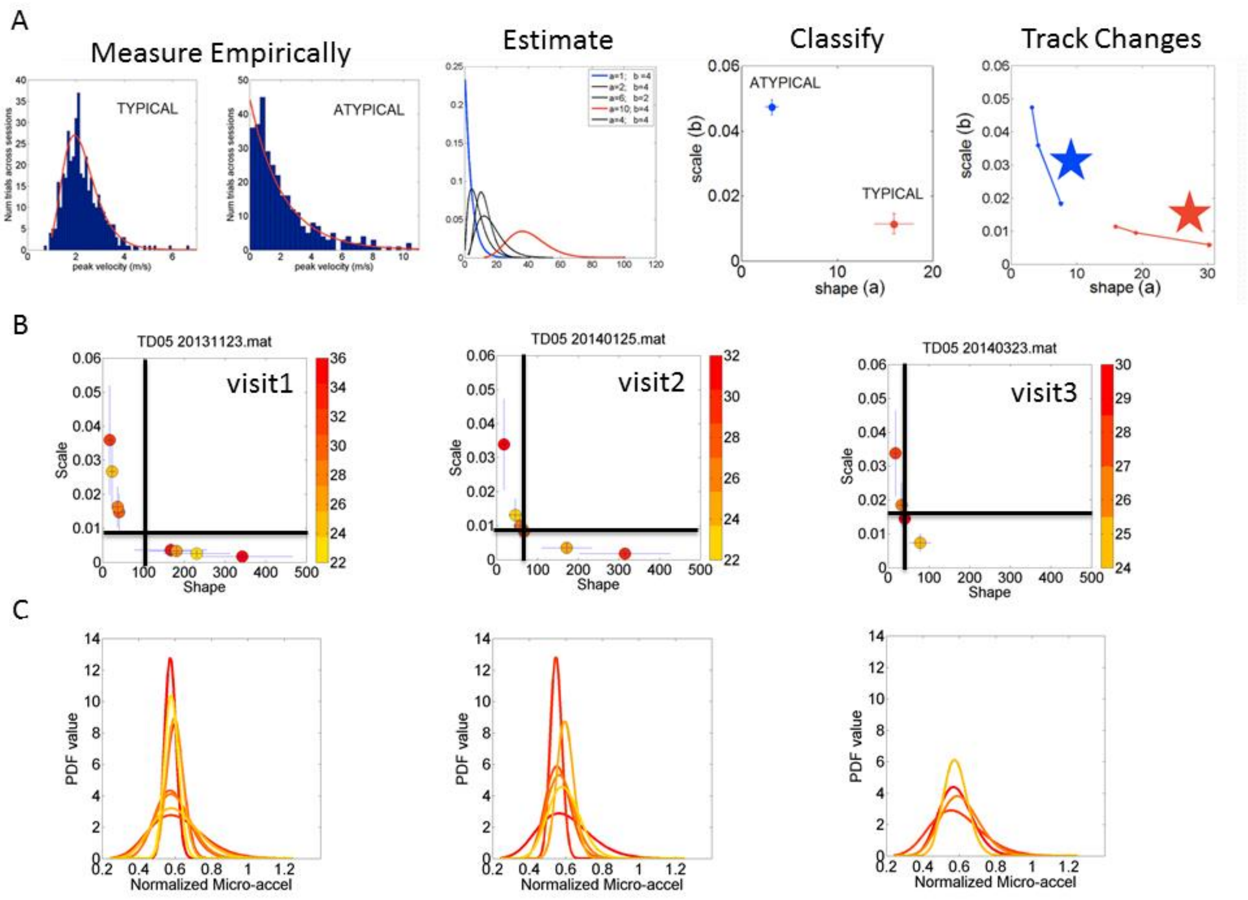

Supplementary Figure 3

Methods of analyses: (A) Schematics of the steps for analyses of fluctuations in motor performance. The first step is to measure the fluctuations in a physiological rhythm, e.g. speed, acceleration, heart rate, temperature, respiration, etc. In this case, they are the linear acceleration or the angular velocity continuously output by the sensors as a time series. The peaks (fluctuation

in amplitude) are gathered into a frequency histogram and the maximal deviations from the empirically estimated mean are then normalized and gathered into a frequency histogram. The probability density function is then estimated using the maximum likelihood estimation (MLE) methods. In this case the continuous Gamma family of probability distributions is a good fit. As such the Gamma process is used to estimate for each minute-block of data the shape and scale parameters, plotted here on the Gamma parameter plane with 95% confidence intervals. The coordinates of the Gamma estimates on the Gamma parameter plane are tracked over time, as they shift along a stochastic trajectory across the 13 hours of recordings. (B) Examples from the three visits of one baby illustrating the steps in (A). Each point on the Gamma plane is plotted with 95% confidence intervals for each of the temperature intervals depicted by the color bar. The median lines are obtained from the median shape and the median scale to divide the Gamma parameter plane into quadrants with the LUQ (high noise and more skewed distributions) vs. RLQ (low noise and less skewed distributions). As before, the color bar depicts the temperature regimes of the most relevant motion (maximal deviations from the mean). (C) The estimated PDFs corresponding to each temperature range in B.

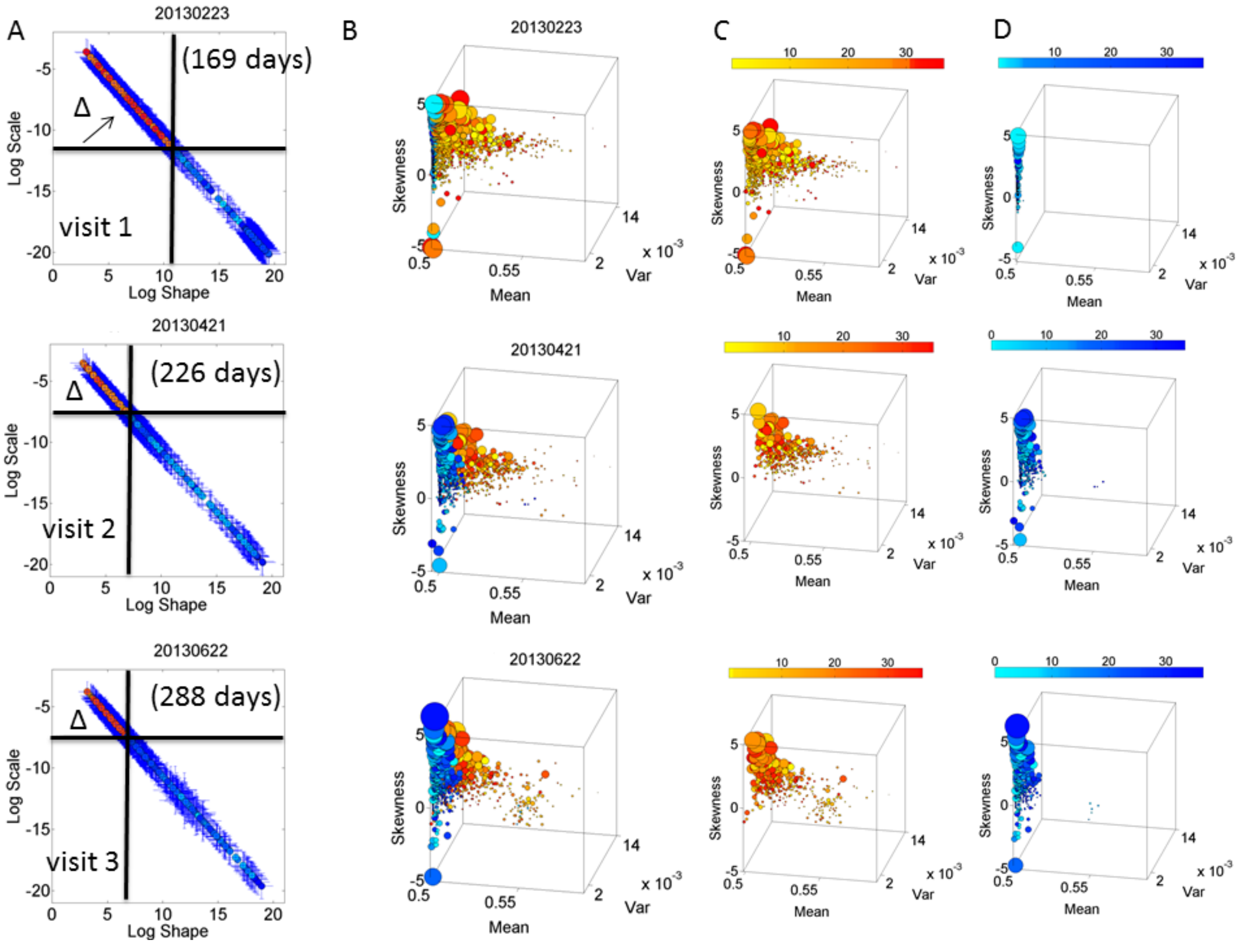

Supplementary Figure 4

Methods used to track longitudinal shifts in noise levels. (A) Sample stochastic maps to quantify the rates of change in noise evolution on the Gamma parameter plane for one (CT) baby across 3 visits spanning 4 months across infancy. Each plot is obtained from the motions registered in one visit with each point representing an estimation of 1,200 measurements (built with one-minute blocks, 20Hz x 60 seconds values registered over the span of 8 hours.) Each colored circle represents the empirically estimated Gamma parameters obtained using MLE, plotted with 95% confidence intervals on the log-log Gamma parameter plane. The color represents the average temperature across the *range* in that minute-block (see color bars on the three-dimensional plots). The noise range is tracked using the median of the values as a cutoff

between higher and lower noise levels. Points above the median scale values (higher noise-to-signal values on the upper-left quadrant) are plotted in shades of red, whereas those in the lower levels of noise (lower-right quadrant) are plotted in shades of blue. (B) Five dimensional scatter plots involving the estimated mean (x-axis), estimated variance (y-axis), the skewness (z-axis) and the kurtosis (the size of the marker) color-coded with blue or red gradients according the median cutoff levels of noise. (C-D) Separable evolution of the high and low noise levels across visits plotted along with their temperature ranges. Darker colors represent higher values of temperature on average for the corresponding minute-block fluctuations in amplitude that went into the Gamma-process estimation. Darker colors of higher temperature on average are from actively generated motions registered by the inertial sensors. These larger motions consume more battery energy and therefore, on average, generate more heat.

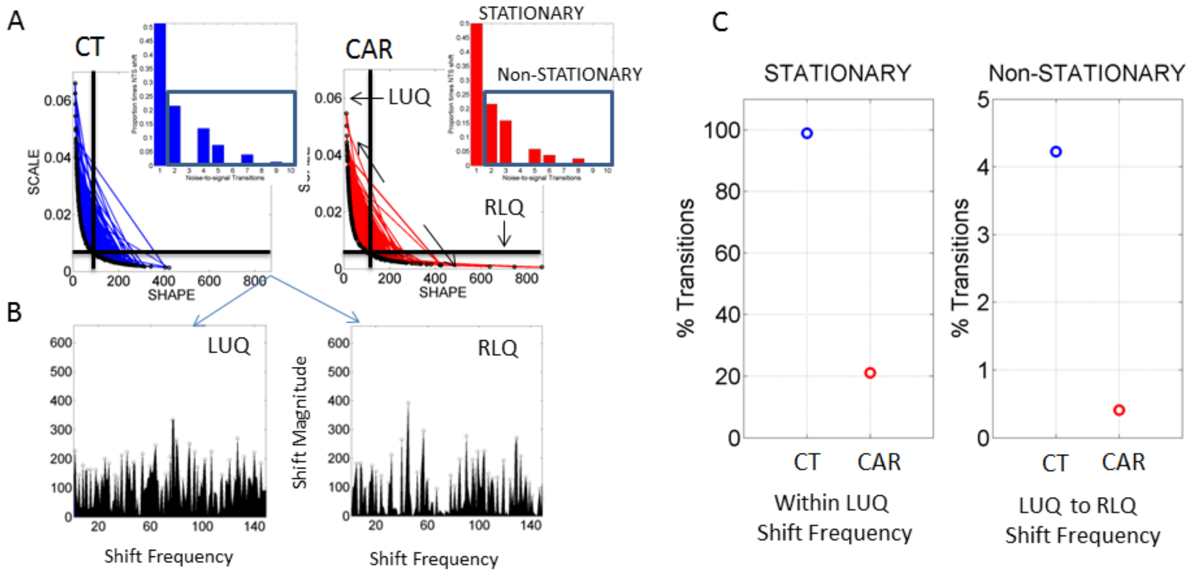

Supplementary Figure 5

Tracking the rate of adaptive change through the noise-to-signal transitions on the Gamma plane. (A) The stochastic trajectory obtained from two representative infants, one CT and one

CAR across 8 hours using 1minute blocks (with ½ minute sliding window) to continuously estimate the Gamma parameters. The median lines obtained from the median shape and median scale values divide the Gamma plane into quadrants whereby the amplitude and frequency of the transition of points between the left upper quadrant (LUQ) and the right lower quadrant (RLQ) are quantified. Arrows mark segments of the trajectories (change in probability distribution) as they transition from the LUQ of high noise and skewed distributions to the RLQ of low noise and near-symmetric distributions. Insets show the histograms with 10 bins quantifying the proportions of stationary (inner-quadrant) transitions (1 transition in first bin) and those quantifying non-stationary (intra-quadrant) transitions (2 or more in subsequent bins) highlighted with a rectangle. (B) Area-plots of the shifts in the trajectory of (A) corresponding to each quadrant of interest for the CT baby. The graphs are obtained by computing the magnitude of the velocity vector connecting the points positioned on the Gamma parameter plane representing the estimated PDF. The peaks are marked in each plot. (C) Percent frequency of the shifts for each representative CAR and CT baby quantified within a given quadrant (the LUQ in this case) and between quadrants (from the LUQ to the RLQ in this case) denoting stationary and non-stationary shifts respectively. These quantities are the averaged percent values across the three visits.

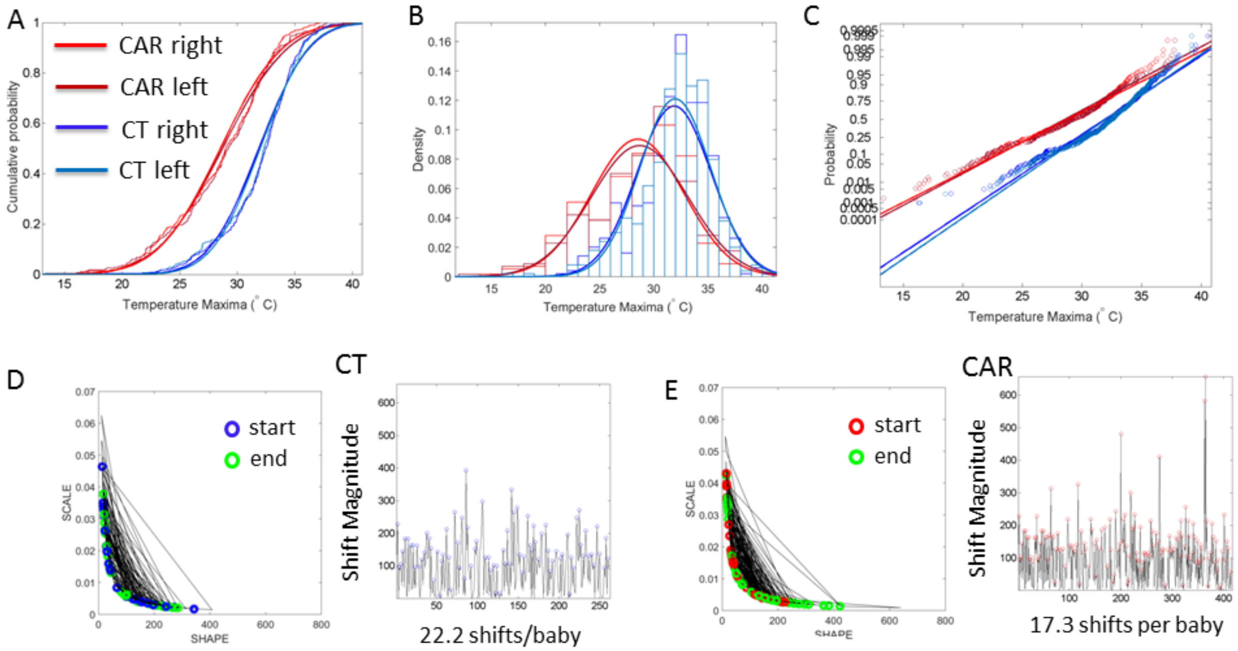

Supplementary Figure 6

Differences between the groups based on clinical labels given from birth status are evident in the temperature and motion data. (A) Empirical cumulative probability distribution (eCDF) plots of the patterns of fluctuations in temperature maxima for the right and left sensors pooled across all babies in the group labeled control (CT) with no complications at birth and all babies in the group labeled clinically at risk (CAR) with complications at birth (significant differences reported in the main text). (B) Same as in (A) showing the probability density plots for each group. (C) Same as in A-B showing the probability plots using the normal distribution as the fitting theoretical probability distribution as the model (no statistically significant differences between right and left patterns of fluctuations in temperature maxima were registered within each group). (D-F) Stochastic trajectories across visits pooled across all babies of a group plotted on the Gamma parameter plane. Corresponding proportions of shifts in parameter values, with far fewer shifts in CAR as a group than those quantified in the CT group.

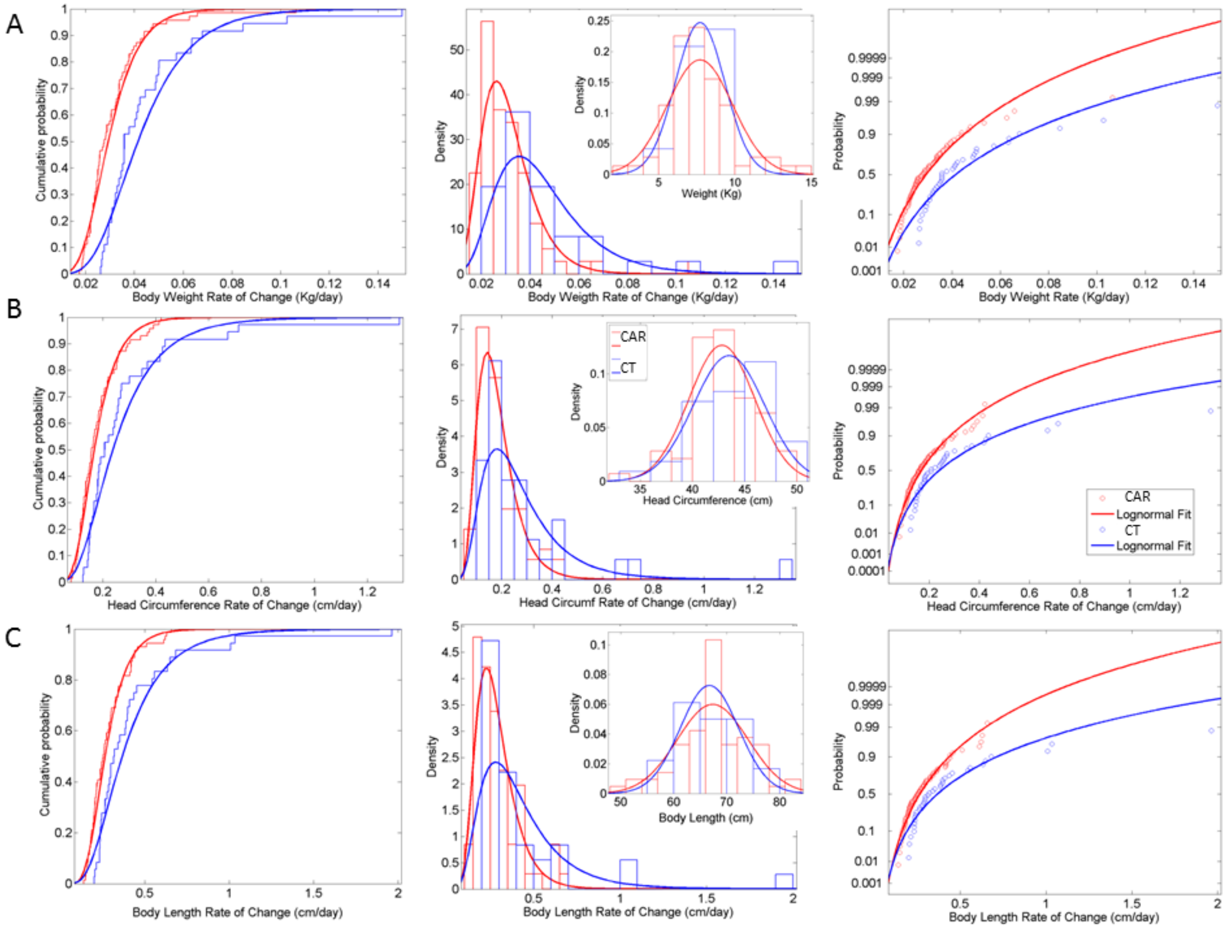

Supplementary Figure 7

Contrasting statistical differences found in physical growth data taken as absolute values *vs.* data taken as incremental (velocity) values representing rates of change differences since birth between the two CT and CAR groups. (A-C) Left-most panel shows the empirical cumulative distributions of body weight, head circumference and body length respectively. Data are taken incrementally as a rate of change from the day of birth until the visit day (all 3 visits data pooled across all CT, blue curve and all data pooled across all CAR babies, red curve). Middle panels contain the same data in probability density format and right-most panels are in probability-plot format. In all the incremental data is well fit by the theoretical lognormal distribution shown by the smooth curve. Notice central panel insets showing the frequency

histograms of the absolute values of physical parameters currently in use by many pediatricians and referenced to growth charts of absolute values from normative data based on the normal distribution (see **Table 3** for further details on statistical comparisons.)
